# Molecular Profiling of Keratinocyte Skin Tumors Links *Staphylococcus aureus* Overabundance and Increased Human β-Defensin-2 Expression to Growth Promotion of Squamous Cell Carcinoma

**DOI:** 10.3390/cancers12030541

**Published:** 2020-02-26

**Authors:** Nandhitha Madhusudhan, Manuela R. Pausan, Bettina Halwachs, Marija Durdević, Markus Windisch, Jan Kehrmann, VijayKumar Patra, Peter Wolf, Petra Boukamp, Christine Moissl-Eichinger, Lorenzo Cerroni, Jürgen C. Becker, Gregor Gorkiewicz

**Affiliations:** 1 Institute of Pathology, Medical University of Graz, Neue Stiftingtalstrasse 6, 8010 Graz, Austria; nandhitha.madhusudhan@medunigraz.at (N.M.); Bettina.halwachs@medunigraz.at (B.H.); marija.durdevic@medunigraz.at (M.D.); markus.windisch@medunigraz.at (M.W.); 2 Theodor Escherich Laboratory for Medical Microbiome Research, Medical University of Graz, Auenbruggerplatz 15, 8036 Graz, Austria; 3 Department of Internal Medicine, Medical University of Graz, Auenbruggerplatz 15, 8036 Graz, Austria; manuela.pausan@medunigraz.at (M.R.P.); christine.moissl-eichinger@medunigraz.at (C.M.-E.); 4 BioTechMed, Interuniversity Cooperation, Mozartgasse 12/11, 8010 Graz, Austria; 5 Institute of Medical Microbiology, University Hospital Essen, University of Duisburg-Essen, Hufelandstrasse 55, 45122 Essen, Germany; jan.kehrmann@uk-essen.de; 6 Department of Dermatology, Medical University of Graz, Auenbruggerplatz 8, 8036 Graz, Austria; vijaykumar.patra@medunigraz.at (V.P.); peter.wolf@medunigraz.at (P.W.); lorenzo.cerroni@medunigraz.at (L.C.); 7 Leibniz Research Institute for Environmental Medicine, Auf’m Hennekamp 50, 40225 Düsseldorf, Germany; Petra.Boukamp@IUF-Duesseldorf.de; 8 Department of Translational Skin Cancer Research and Department of Dermatology, German Cancer Consortium (DKTK), University Hospital Essen, Universitätsstrasse 1, 45141 Essen, Germany

**Keywords:** skin microbiota, actinic keratosis, squamous cell carcinoma, basal cell carcinoma, *Staphylococcus aureus*, antimicrobial peptides, human β-defensin

## Abstract

The skin microbiota plays a prominent role in health and disease; however, its contribution to skin tumorigenesis is not well understood. We comparatively assessed the microbial community compositions from excision specimens of the main human non-melanoma skin cancers, actinic keratosis (AK), squamous cell carcinoma (SCC) and basal cell carcinoma (BCC). Keratinocyte skin tumors are characterized by significantly different microbial community compositions, wherein AK and SCC are more similar to each other than to BCC. Notably, in SCC, which represents the advanced tumor entity and frequently develops from AK, overabundance of *Staphylococcus aureus,* a known skin pathogen, was noted. Moreover, *S. aureus* overabundance was significantly associated with increased human β-defensin-2 (hBD-2) expression in SCC. By challenging human SCC cell lines with *S. aureus*, a specific induction of hBD-2 expression and increased tumor cell growth was seen. Increased proliferation was also induced by directly challenging SCC cells with hBD-2. Together, our data indicate that a changed microbial community composition in SCC, specified by *S. aureus* overabundance, might promote tumor cell growth via modulation of hBD-2 expression.

## 1. Introduction

Keratinocyte skin tumors are represented by two main entities, cutaneous squamous cell carcinoma (SCC) and basal cell carcinoma (BCC), which account for more than 90% of all skin malignancies with suggested 2–3 million global cases per year [1,2,3]. The main risk factor for these tumors is exposure to ultraviolet radiation. Actinic keratosis (AK) is a premalignant lesion typically developing on photo-damaged skin. It can progress into SCC, but it can also regress or remain stable over a long time without getting malignant. Thus, environmental factors other than UV also seem to influence keratinocyte tumor development. Notably, the incidence of SCC is increased among immunosuppressed individuals (e.g., organ transplant recipients), and it is suggested that microbes such as the human papilloma virus (HPV) might trigger tumor development [4,5]. Human skin harbors a diverse, highly individualized, and site-specific microbiota, consisting of bacteria, fungi, and virus whose contribution to skin tumor development is not well understood [6,7,8]. During carcinogenesis, skin morphology and metabolism are significantly changed. Consequently, the microbial habitat is changed which might lead to an altered microbial colonization potentially favoring pro-tumorigenic microbes, which in turn could perpetuate tumorigenesis. Such an example was recently shown in colorectal carcinogenesis, wherein overexpression of a specific bacterial lectin (Gal-GalNAc) on the neoplastic colon epithelium leads to selective enrichment of tumor-driving *Fusobacterium nucleatum* [9]. Notably, bacteria can contribute to neoplastic processes in various ways, which include the production of genotoxins leading to DNA damage or their ability to influence the tumor microenvironment such as the immune system in the vicinity of the tumor [10,11]. Indeed, it was shown recently that in an animal model of keratinocyte skin cancer due to chronic inflammation, tumor formation was dependent on bacterial infection and was specifically driven by flagellin (the ligand for TLR-5), whereas antibiotic treatment inhibited tumor formation [12]. Antimicrobial peptides (AMPs), innate immune factors produced by epithelia and immune cells, are of special interest in this context, since they are induced in response to specific microbes and are also implicated in tumorigenesis, since they affect tumor cell growth and migration—both are features associated with tumor progression [13,14]. 

To gain insights into the contribution of the bacterial microbiota to skin tumors we comparatively profiled microbial compositions of AK, SCC, and BCC cases using excised tumor specimens containing their whole microbiota at site. We identified significantly different microbial communities in the different tumor entities. The most prominent finding was overabundance of *Staphylococcus aureus* in SCCs. Importantly, *S. aureus* overabundance was strongly associated with increased expression of the AMP human β-defensin-2 (hBD-2) in SCCs. Consequently, challenge of human cutaneous SCC cells with *S. aureus* showed a specific induction of *hBD-2* expression and also increased tumor cell proliferation. 

## 2. Results

### 2.1. Microbial Colonization of Keratinocyte Skin Tumors

**Figure 1:**
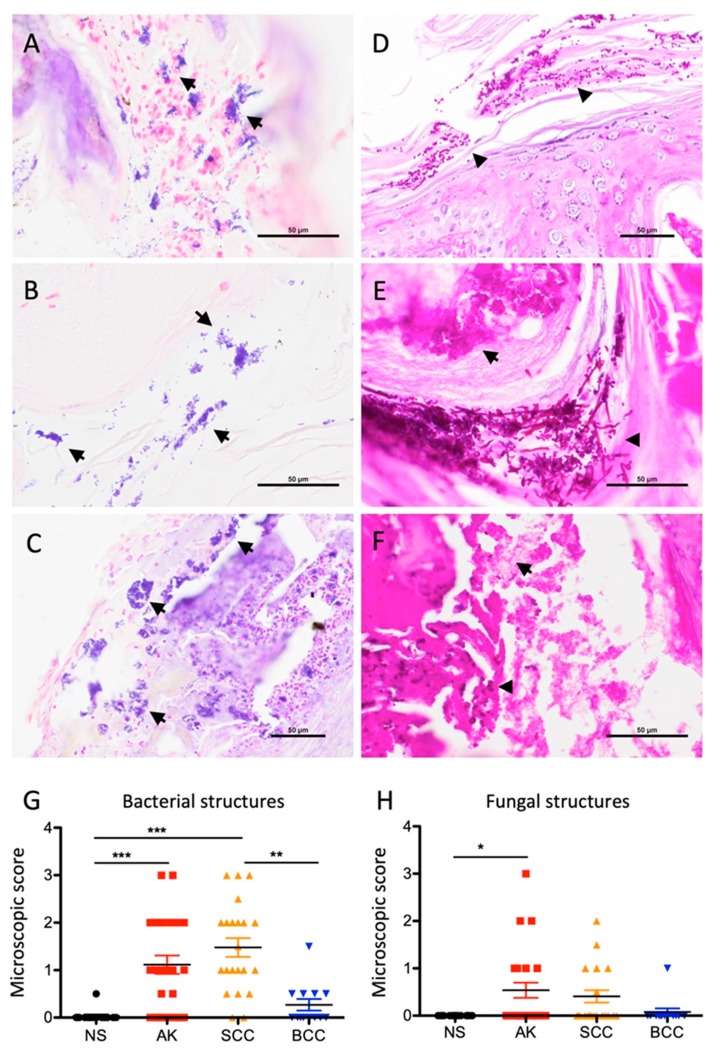
Abundant microbial structures are prevalent in actinic keratosis (AK) and squamous cell carcinoma (SCC). (**A**–**F**) Bacterial (arrows) and fungal (arrow heads) structures in AK (**A**,**D**,**E**) and SCC (**B**,**C**,**F**) specimens (**A**–**C**, Gram stain; **D**–**F**, PAS stain). (**G**) Microscopic scoring indicates significantly increased bacterial structures in AK and SCC compared to basal cell carcinoma (BCC) and NS (normal skin) samples (** *p* < 0.01; *** *p* < 0.005; Kruskal–Wallis test; Dunn’s multiple comparison test). (**H**) Slightly increased fungal structures in AK samples (* *p* < 0.05; Kruskal–Wallis test; Dunn’s multiple comparison test).

We assessed 88 skin samples originating from sun exposed skin sites (head, neck, and upper extremity) representing excised tissue specimens (total excisions, *n* = 54; punch biopsies, *n* = 24; shave biopsies, *n* = 10). In addition to the tumor samples (13 BCCs, 25 AKs, and 22 SCCs), 28 skin samples without neoplasia were included as controls, including healthy skin, non-neoplastic skin adjacent to tumors, and samples from psoriasis cases (a detailed sample designation is given in Table S1). Skin undergoes significant changes in histology during neoplasia development leading to an altered microbial habitat thereby potentially influencing microbial colonization. AKs and SCCs are often covered with abundant lamellar keratin on the lesional surface (termed hyper- and parakeratosis), which is a less pronounced feature in BCC (Figure S1A–F). Microscopy revealed numerous microbial structures in AKs and SCCs often associated with hyper- and parakeratosis, whereas in BCC and normal skin, microbial structures were rarely detected (Figure 1A–F). Microbial structures resembled bacteria and to lesser extent also fungi, which were microscopically differentiated based on morphology and staining behavior (see materials and methods for details). Semi-quantitative scoring of microbial structures indicated significantly increased bacteria in AKs and SCCs compared to BCCs and normal skin (Figure 1G). Bacterial structures were not only present on the surface of AK and SCC cases, but were also present within invasive areas of the tumors (Figure S2A,B). In addition, fungi were slightly increased in AKs (Figure 1H).

### 2.2. Distinct Microbial Community Types Are Prevalent in Keratinocyte Skin Tumors, wherein AK and SCC Are Characterized by Staphylococcus Overabundance

**Figure 2:**
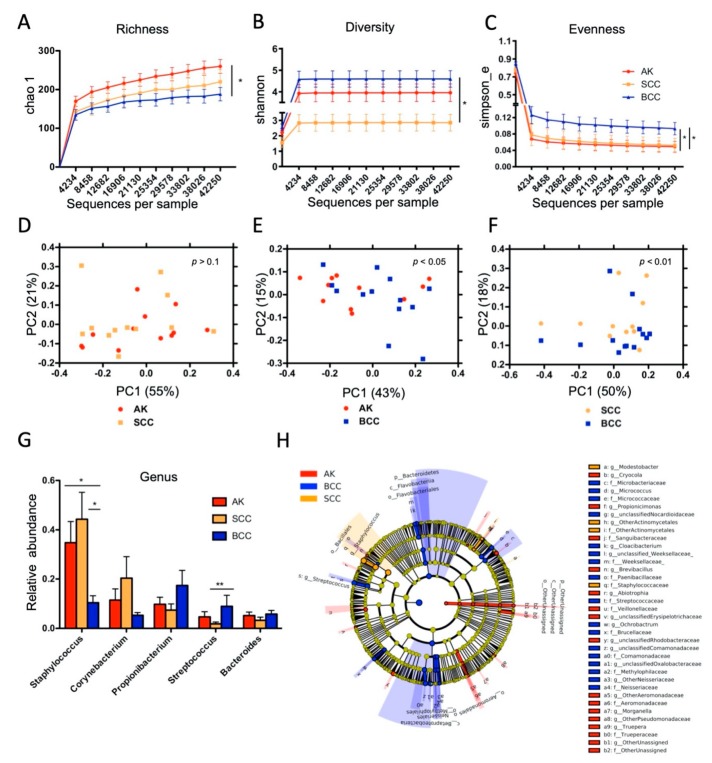
Different microbial community types in keratinocyte skin tumors. (**A**) Microbial richness (Chao1) was significantly higher in AK compared to BCC and (**B**) diversity (Shannon) was significantly lower in SCC compared to BCC. (**C**) Evenness (Simpson) was significantly lower in AK and SCC compared to BCC (unpaired *t*-test; * *p* < 0.05). (**D**–**F**) Principal coordinate analysis indicates significantly different microbial communities in AK and SCC compared to BCC (measure: weighted unifrac; ANOSIM). (**G**) Relative abundance of *Staphylococcus* was significantly higher in AK and SCC compared to BCC (* *p* < 0.05), whereas relative abundance of *Streptoccocus* was significantly increased in BCC compared to SCC (** *p* < 0.01; Kruskal–Wallis). (**H**) LEfSe analysis specifying taxa with increased relative abundance in tumors. Note the association of *Staphylococcus* (LDA > 5) with SCC, as well as *Streptococcus* (LDA > 4) and *Betaproteobacteria* (LDA > 4) with BCC (a comprehensive bar-chart and tabular representation of the LEfSe output is given in Figure S5 and Table S3; a higher magnification of Figure 2H is given as Figure S6).

We performed 16S rRNA gene-based microbial community profiling in a subset of AK (*n* = 12), SCC (*n* = 12), and BCC (*n* = 13) samples to assess the taxonomic composition of the tumor-associated microbes. In addition, 8 normal skin specimens were included in the analysis (see Table S1 for sample details). The variable V1-2 region of the bacterial 16S rRNA gene was amplified by PCR and products were resolved on an Illumina^TM^ Miseq next generation sequencer (Table S2). A total of 17,747,248 reads were generated, corresponding to 199,630 ± 132,100 reads per sample on average. After quality filtering, denoising, and clustering, 117 ± 37 operational taxonomic units (OTU97%ID) were allocated per sample on average. Alpha-diversity measures were significantly different between healthy skin and all keratinocyte tumors (Figure S3A–C). In tumors, microbial richness was significantly increased in AK compared to BCC and diversity was significantly lower in SCC compared to BCC. Evenness was significantly lower in AK and SCC compared to BCC (Figure 2A–C). The latter finding might indicate that certain microbes are dominating the community in AK and SCC, thereby leading to an uneven community composition. Principle coordinate analysis (PCoA; measure: unifrac) identified significantly different microbial communities in all four sample types (Figure S3D,E). When PCoA was performed in a pair-wise fashion on tumor samples, significantly different microbial communities in AK and SCC compared to BCC were seen, whereas no significant difference was evident between AK and SCC samples, indicating that AK and SCC are generally more similar in their microbial community composition (Figure 2D–F). 

We next determined which microbes are specifically associated with tumors. At genus level relative abundance of *Staphylococcus* was significantly increased in AKs and SCCs, while relative abundance of *Streptococcus* was significantly increased in BCCs (Figure 2G and Figures S3F and S4). Comparative analysis with the linear discriminant analysis effect size analysis (LEfSe) confirmed increased relative abundance of *Staphylococcus* in SCCs (LDA > 5; 50.07 ± 14.43%), which was also the dominant taxon in AKs (38.62 ± 14.05%), whereas *Streptococcus* (LDA > 4; 17.42 ± 10.51%) was increased in BCCs (Figure 2H and Figure S5). LEfSe analysis revealed also other differentially abundant taxa, associated mainly with BCCs, like *Betaproteobacteria* and *Bacteroidetes*, although with overall lower relative abundance. Normal skin showed significantly increased *Streptomyces* compared to keratinocyte tumors in the LEfSe analysis (Figures S3G and S7). A complete listing of taxa revealed by LEfSe analysis are shown in Tables S3 and S4. 

**Figure 3:**
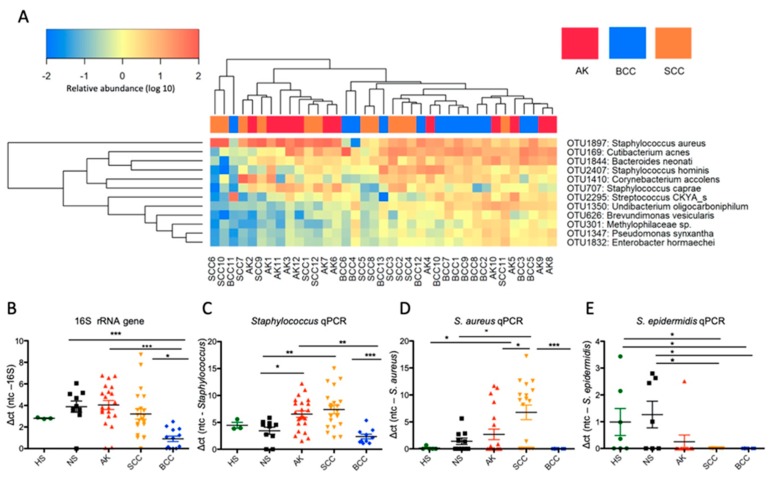
Dominant taxa, bacterial, *Staphylococcus* and *S. aureus* loads in keratinocyte skin tumors. (**A**) Heat map representation of dominant taxa revealed by unsupervised hierarchical clustering of log(10)-transformed data indicates a predominant but not exclusive association of *S. aureus* with AK and SCC samples. Species level taxonomy was assigned by using the EzBioCloud database [15]; see also Figure S5 for a complete heat map representation. Analysis of an expanded sample set by means of qPCR identifies that (**B**) bacterial load (16S rRNA gene) is significantly reduced in BCC, (**C**) *Staphylococcus* (genus) loads are significantly increased in AK and SCC, and (**D**) *S. aureus* loads are significantly increased in SCC. (**E**) *S. epidermidis* loads are significantly reduced in tumors (B and E: Kruskal–Wallis test; Dunn’s multiple comparison test; C and D: ANOVA; Turkey’s multiple comparison test; * *p* < 0.05; ** *p* < 0.01; *** *p* < 0.005).

We next employed unsupervised hierarchical clustering of tumor samples, wherein we assigned OTUs to species level taxonomy by using the EzBioCloud database [15]. This analysis indicated a highly individualized microbiota composition for the different specimens; nevertheless, a strong but not exclusive association of *Staphylococcus aureus* with AK and SCC samples was observed (Figure 3A, Figure S9). Interestingly, when *S. aureus* was abundant, other typical skin microbes like *Cutibacterium* (formerly *Propionibacterium*) *acnes* were often low, indicating a possible competitive exclusion between these taxa. To confirm the dominant colonization of AKs and SCCs with *S. aureus* and to measure the microbial load, we next employed quantitative PCR (qPCR) within an increased sample set. These analyses indicated that bacterial load was not changed in AKs and SCCs compared to normal skin; however, it was significantly reduced in BCCs. *Staphylococcus* (genus) loads were significantly increased in AKs and SCCs, whereas *S. aureus* loads were significantly increased only in SCCs (Figure 3B–D). Notably, *S. aureus* loads were also significantly increased from adjacent non-lesional skin (NS) to neoplastic skin in paired samples (Figure S10). Interestingly, *S. epidermidis*, a proposed antagonist of *S. aureus* [16], was significantly reduced in tumors pointing again to a possible competition with *S. aureus* (Figure 3E). Notably, chronic plaque psoriasis samples showed no increased *Staphylococcus* and *S. aureus* loads in qPCR analyses (Figure S11). Thus, *Staphylococcus* and *S. aureus* overabundance in AK and SCC might not be a sole phenomenon driven by increased amounts of keratin on the tumor surface, since psoriasis is also characterized by a vigorous hyper- and parakeratosis.

Collectively, these data indicate significantly different microbial community types in keratinocyte skin tumors, which deviate from normal skin. AKs and SCCs show an uneven community composition wherein the taxon *Staphylococcus* dominates, and in SCCs, *S. aureus* is the most abundant taxon. This change in microbial community is linked to a reduction of typical skin commensals like *S. epidermidis* or *C. acnes*. BCCs show a reduced bacterial load and represent a more variable community composition.

### 2.3. S. aureus Is Associated with Hyperkeratotic Regions in AK and SCC and Is Found in Areas with Invasive Tissue 

**Figure 4:**
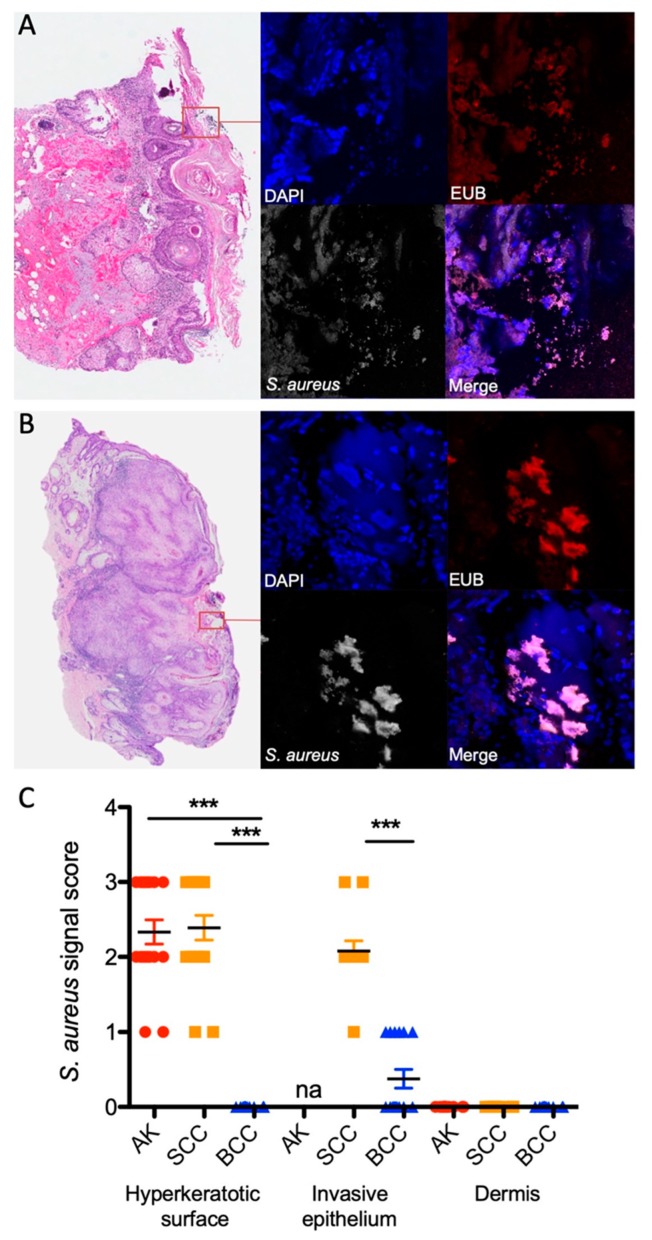
*S. aureus* is associated with hyperkeratotic areas in AK and SCC and invasive tumor in SCC. In situ visualization of *S. aureus* in the hyperkeratotic layer in AK (**A**) and SCC (**B**) using FISH (DAPI: blue, EUB338: red; *S. aureus*: grey; magnification 650×). Representative samples are shown (left: H&E stain; right: FISH). (**C**) Semi-quantitative scoring of *S. aureus* specific signals in hyperkeratotic surface areas and invasive tumor areas in a sub-set of specimens (*** *p* < 0.005; Kruskal Wallis; na, not applicable; AK, *n* = 5; SCC, *n* = 5; BCC, *n* = 5).

To investigate the in situ localization of *S. aureus* in lesions, we employed fluorescence in situ hybridization (FISH) using a panbacterial (EUB) and an *S. aureus*-specific probe within a sub-set of tumor samples. For this analysis, we chose samples with overall high *S. aureus* loads (determined by qPCR) to facilitate enough signal strength for FISH detection (Table S1). A significantly increased number of *S. aureus*-specific signals were scored in the hyperkeratotic surface areas of AK and SCC samples, as well as in invasive sites of SCCs. Only sparse signals were detected in BCC samples, and no bacterial signals could be detected in the dermis (stroma) of any of the samples (Figure 4A–C). These findings strongly suggest that *S. aureus* colonizes the hyperkeratotic surface areas of AKs and SCCs as well as invasive tumor tissue of SCCs. 

### 2.4. Antimicrobial Peptide Expression Is Changed in Keratinocyte Skin Tumors, and hBD-2 Expression Correlates Significantly with S. aureus Loads in SCC

**Figure 5:**
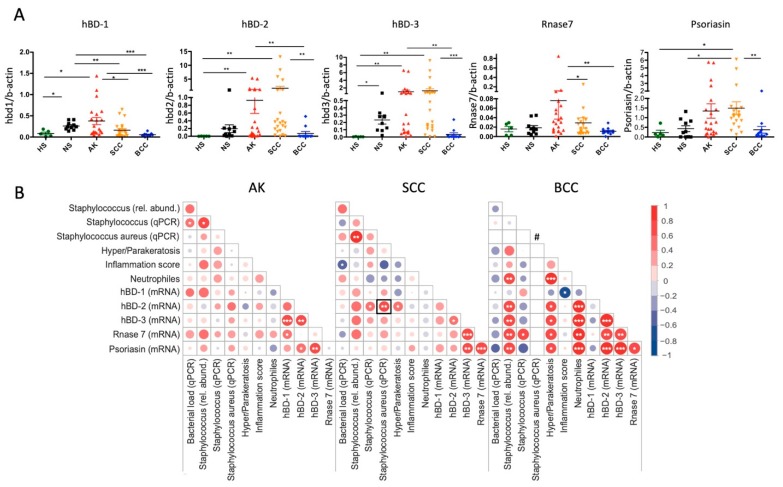
Antimicrobial peptide mRNA expressions and feature correlation analysis. (**A**) Transcription levels of *hBD-1* and *Rnase 7* were significantly increased in AK. A marked increase of *hBD-2, hBD-3*, and to a lesser extent also of *psoriasin* mRNA was found in SCC and AK compared to BCC and normal skin (* *p* < 0.05, ** *p* < 0.05, *** *p* < 0.005, Kruskal–Wallis; HS, *n* = 5; NS, *n* = 10; AK, *n* = 21; SCC, *n* = 22; BCC, *n* = 11; data are mean ± SEM). (**B**) Pearson correlation matrix of assessed features in AK, SCC, and BCC. Strong correlations are indicated by large circles, whereas weak correlations are indicated by small circles. The color of the scale bar represents the nature of correlation with 1 indicating a strong positive correlation (dark red) and -1 representing a strong negative correlation (dark blue). *t*-tests to the individual correlations were applied for significance testing and significant correlations were indicated with * *p* < 0.05, ** *p* < 0.05, *** *p* < 0.005; shown as stars in the respective circles). The significant correlation of *S. aureus* loads with *hBD-2* mRNA expression in SCC is highlighted (box); # denotes that no *S. aureus* signals were detected in BCC with qPCR.

While hBD-1 is constitutively expressed in skin, hBD-2, hBD-3, RNase7, and psoriasin (*S100A7*) can be modulated by microbes [17,18]. Notably, AMPs could also modulate epithelial cell growth and migration [19,20,21]. To assess a potential interdependence between microbial colonization and AMP expressions, we measured AMP mRNA levels in our samples via qRT-PCR. Transcription levels of *hBD-1* and *Rnase7* were significantly increased in AKs, although at relatively low levels. Notably, a marked increase of *hBD-2, hBD-3* and to a lesser extent also of *psoriasin* mRNA was found in SCCs and AKs, as compared to BCCs and normal skin (Figure 5A). We then performed a broad correlation analysis of assessed parameters including AMP expressions; bacterial, *Staphylococcus*, and *S. aureus* loads; and histologically scored levels of hyper-/parakeratosis, the inflammatory infiltrate, and neutrophilic granulocytes as known predictors of skin tumor progression [22]. As shown in the correlation matrices in Figure 5B, several significant correlations were found. Most relevant to our survey, we found a significant positive correlation of *hBD-2* expression with *Staphylococcus* and *S. aureus* loads in SCCs. In addition, significant positive but differing correlations within AMP mRNA levels were evident in the entities. Only *hBD-3* showed a significant positive correlation with *hBD-2* and *psoriasin* expression consistently in all three entities together. No clear correlations between hyper- and parakeratosis, inflammation (measured as density of inflammatory cells in the dermis), and neutrophilic granulocytes were evident. Only in BCCs, hyper-/parakeratosis and neutrophiles were associated with increased AMP expressions. Two significant negative correlations were found: inflammation with *hBD-1* expression in BCCs and inflammation with bacterial load in SCCs. Together, this analysis suggests a possible connection between the type of microbial colonization and AMP expressions with particular emphasis on *S. aureus* overabundance and *hBD-2* expression in SCCs. In a more general sense, our data may suggest that an altered microbial colonization in keratinocyte skin tumors might influence AMP expressions with potential downstream effects on tumor cell biology.

### 2.5. S. aureus Challenge Induces hBD-2 mRNA Expression and Stimulates Growth of Cutaneous SCC Cells

**Figure 6:**
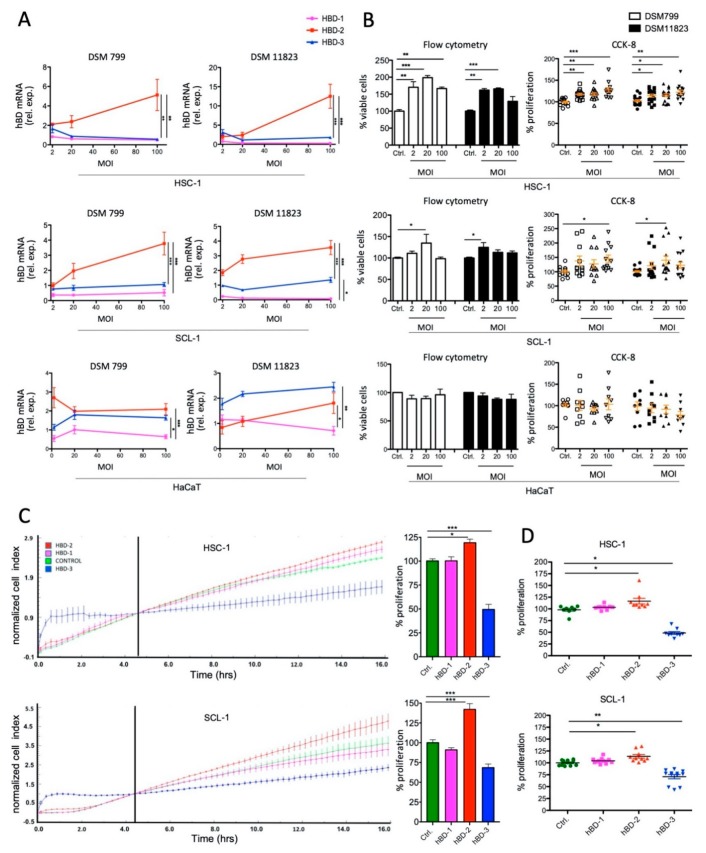
*S. aureus* challenge of cutaneous SCC cells stimulates *hBD-2* expression and induces cell proliferation. (**A**) HSC-1, SCL-1, and HaCaT cells challenged with increasing MOIs of *S. aureus* DSM799 and DSM11823 show a dominant and dose-dependent increase of *hBD2* mRNA expression in SCC cells measured by qPCR (* *p* < 0.05; ** *p* < 0.01; *** *p* < 0.005, one-way ANOVA, Bonferroni correction). (**B**) Significantly increased viable SCC cell numbers due to *S. aureus* challenge as compared to controls assessed by flow-cytometry (* *p* < 0.05; ** *p*< 0.01; *** *p*< 0.005, one-way ANOVA; Dunnett’s multiple comparison test) and the CCK-8 assay (* *p* < 0.05; ** *p* < 0.01; *** *p* < 0.005, Kruskal–Wallis, Dunn’s multiple comparison test). HaCaT cells show no increase in cell numbers. (**C**) Real-time monitoring of HSC-1 (top) and SCL-1 (bottom) cell growth with the xCelligence^TM^ system during challenge with 20 μg/mL of hBD-1, -2 and -3, respectively. An increased cell proliferation due to hBD-2 challenge and a decreased proliferation due to hBD-3 challenge was detected after 16 h (measure: normalized cell index; representative graph of three repetitions). Bar charts on the right indicate relative (%) cell proliferation compared to controls at 16 h (* *p* < 0.05; *** *p* < 0.005, one-way ANOVA, Turkey’s multiple comparison test). (**D**) hBD-2 increases and hBD-3 decreases proliferation of HSC-1 (top) and SCL-1 (bottom) cells treated with 20 μg/mL AMPs for 24 h measured by the CCK-8 assay (* *p* < 0.05; ** *p* < 0.01, Kruskal–Wallis, Dunn’s multiple comparison test).

To investigate whether *S. aureus* leads to induction of hBDs in SCC cells and whether this effect might stimulate tumor cell growth, we performed co-culture experiments with *S. aureus* and SCC cell lines originating from cutaneous SCCs, as well as HaCaT cells resembling non-tumorous keratinocytes. We used two *S. aureus* strains (DSM 799 and DSM 11823) with increasing MOIs of 2, 20, and 100, respectively. Of note, these strains are devoid of classical exotoxins, a necessity for co-culture, since a toxin producing strain (DSM 1104 harboring enterotoxin G and I) induced immediate cell death in preliminary experiments (not shown). *hBD-1, -2* & *-3* mRNA expressions were measured by qRT-PCR after 24 h challenge, and bacterial growth was controlled simultaneously by CFU plating (Figure S12A–C). Challenge with both strains led to a significantly increased expression of *hBD-2* compared to *hBD-1* and *-3* in a dose-dependent manner in both SCC cell lines, whereas HaCaT cells showed no or only a minor increase of both *hBD-2* and *-3* mRNA (Figure 6A). We also assayed cell growth by measuring viable cells using flow cytometry (Figure S13A–F) as well the colorimetric cell counting kit (CCK-8). Challenge with both *S. aureus* strains caused increased viable cells in a dose-dependent manner in both SCC cell lines in both read-outs, although the growth-promoting effect leveled off to some extent at higher MOIs. No increase was observed by challenge of HaCaT cells (Figure 6B). Subsequently, we challenged SCC cells directly with hBDs (20 µg/mL, [13]) to monitor the net AMP effect on SCC cell proliferation. In these experiments a live-cell growth monitoring system (xCelligence^TM^) in addition to the CCK-8 assay was used. hBD-2 treatment significantly increased proliferation of HSC-1 cells (normalized cell index [nci] 3.147 ± 0.09 vs. 2.64 ± 0.05 at 16 h incubation) and SCL-1 cells (nci 6.28 ± 0.6 vs. 4.38 ± 0.3), whereas hBD-3 significantly impaired cell growth of both HSC-1 (nci 1.41 ± 0.15 vs. 2.64 ± 0.05) and SCL-1 cells (nci 2.96 ± 0.3 vs. 4.38 ± 0.3), respectively. hBD-1 showed no effect on cell growth (Figure 6C). Similar results were obtained when using the CCK-8 assay to measure proliferation at 24 h treatment. HSC-1 cells treated with hBD-2 showed increased growth (0.91 ± 0.02% vs. 0.76 ± 0.05%) and when treated with hBD-3 reduced growth (0.36 ± 0.02% vs. 0.76 ± 0.05%) compared to controls; SCL-1 cells treated with hBD-2 also showed increased growth (0.89 ± 0.02% vs. 0.77 ± 0.01%) and when treated with hBD-3 reduced growth (0.55 ± 0.03% vs. 0.77 ± 0.01%) compared to controls (Figure 6D). Together, these data show that *S. aureus* specifically induces *hBD-2* expression in both cutaneous SCC cell lines, which also led to increased tumor cell growth. Such effects were not seen by challenging the non-tumorous HaCaT cell line. Notably, hBD-2 and hBD-3 have opposing effects on SCC cell growth, wherein the former induces and the latter inhibits proliferation. Since *S. aureus* mainly induces *hBD-2* expression in SCC cells, it is intriguing to speculate that *S. aureus* overabundance in SCC might favor increased tumor cell proliferation which might impact tumor growth and disease progression.

## 3. Discussion

In our study, we investigated the microbiota of the main keratinocyte skin tumors, and histology demonstrated that microbial colonization was a prominent feature mainly of AKs and SCCs. Microbiota profiling showed significantly different microbial community compositions in keratinocyte skin tumors compared to normal skin and also between tumors, although AKs and SCCs showed overall more similarities. Bacterial load was not changed in AKs and SCCs compared to normal skin but was reduced in BCCs. Taxonomic analysis identified increased *Staphylococcus* in AKs and SCCs and overabundance of the species *S. aureus* in SCCs as a prominent feature. *S. aureus* overabundance was also significantly linked to increased *hBD-2* expression in SCC samples. Given the known involvement of AMPs as possible tumor promoters, we subsequently modeled *S. aureus* interactions with cutaneous SCC cells in co-culture experiments, which confirmed a specific induction of *hBD-2* expression by *S. aureus* challenge, leading also to increased tumor cell proliferation. Finally, direct challenge of cutaneous SCC cells with hBD-2 confirmed the growth promoting action of this AMP. Thus, our data suggests a possible link between an altered microbiota in keratinocyte skin tumors influencing AMP expressions, which might promote tumor cell growth and potentially influences tumor progression.

An estimated 15% of human cancers are supposed to have an infectious source [23], wherein inflammation seems to play a key role in triggering the pathologic processes of tumorigenesis [24]. The best characterized example of a bacterial carcinogen is *Helicobacter pylori* inducing chronic gastritis and stomach cancer [25]. *Staphylococcus* species are abundant commensals on human skin, and their preferred sites are areas of high humidity, like the navel, inguinal, and gluteal crease, or feet [26]. The species *S. aureus* could be found in up to 80% of humans, typically colonizing the anterior nares, either persistently or transiently [27]. However, if present on skin, this species is considered pathogenic by driving inflammation [28,29]. As an example, *S. aureus* triggers flares in atopic dermatitis [30,31] and could be frequently isolated from chronic wounds and burn scars [32]. The reason for the increase of *S. aureus* (in relation to the resident microbiota) in cutaneous SCCs is so far unknown but may be due to several reasons. Notably, our findings support two recent publications identifying increased *S. aureus* in cutaneous SCCs [33,34]. Microscopically we identified abundant cocci often in areas of hyper- or parakeratosis. However, *S. aureus* loads determined by qPCR and the amount of hyper-/parakeratosis did not significantly correlate in our study. From the perspective of microbiota–host interdependence, it is possible that a changed microbial habitat, like hyperkeratosis, changes the ability of certain microbes to colonize. Notably, hyperkeratosis per se does not seem to favor *S. aureus* growth, since the hyperkeratotic lesion seborrheic keratosis ("senile wart") does not exhibit increased *S. aureus* loads [33], and analysis of psoriasis samples in our study, which is also hyper- and parakeratotic, showed also no *S. aureus* increase. The latter finding fits into the current knowledge of microbial community changes in psoriasis, wherein lesions seem to be dominated by taxa like *Streptococcus*, *Corynebacterium*, or the fungi *Malassezia* and *Candida* [35,36,37,38,39,40], whereas the involvement of *S. aureus* in this skin diseases is questionable [41]. It seems that *S. aureus* does not infect the skin of immunocompetent individuals unless the skin barrier is injured or broken [42]. Thus, the ulcerating nature of SCC might favor *S. aureus* colonization. Notably, we identified *S. aureus* also in the infiltrative neoplastic epithelium in SCCs, which might be of great relevance for disease progression. In analogy, *F. nucleatum* was recently shown to migrate with invasive and even metastatic tumor tissue in colorectal cancer, thereby directly influencing cancer cell proliferation and tumor growth [43]. Finally, it could be that a changed metabolism of the neoplastic cells might favor *S. aureus* growth. AKs and SCCs are supposed to be impaired in sebum production [34]. This might inhibit commensals like *Propionibacterium*, which are dependent on lipids derived from sebum [7,44], and potentially opens niches for *S. aureus* to proliferate. Interestingly, we found evidence for competitive exclusion between *S. aureus* and *P. acnes* in SCCs and AKs which was also suggested recently [34]. In addition, *S. epidermidis*, a known competitor of *S. aureus*, was also found to be reduced in tumors, and this bacterium was recently shown to protect against skin cancer [8,16,45]. The skin microbiota shows a high degree of individualization; thus, it is possible that other so far unrecognized skin microbes might be associated with skin tumors. Indeed, we did not find dominant *S. aureus* in each SCC case. Consequently, skin fungi might also be involved, since we detected at least histologically increased fungal structures in certain AK samples. Interestingly, fungi could also potentially protect from pathogenic bacteria, as the yeast *Malassezia,* which is supposed to compete against *S. aureus* and which was shown to be reduced in cutaneous SCCs [34]. Together this knowledge might open the avenue for the development of biotherapeutics for out-competition of skin pathogens such as *S. aureus*. Since *S. aureus* is also genetically diverse, showing a varying repertoire of virulence factors, it might also be that only certain strains of *S. aureus* are involved in SCC pathogenesis. Therefore, cultivation studies enabling genomic investigations of *S. aureus* strains derived from AK and SCC samples are important future research aims. Such knowledge might also allow for the development of (bacterial) biomarkers for risk assessment of AK to SCC progression.

Keratinocytes actively contribute to the immune response of the skin by production of various cytokines, chemokines and AMPs. The latter represent essential innate defense molecules, acting against a broad spectrum of microbes [46], and their altered expression in keratinocyte tumors is now intensively studied [47]. Several recent publications employing human primary (foreskin-derived) keratinocytes or HaCaT cells (derived from adult trunk skin) showed upregulation of the AMPs hBD-2, hBD-3, and RNase 7 due to *S. aureus* challenge [17,18,48,49,50,51,52]. However, the differentiation state of the keratinocyte greatly changes AMP expression patterns [53]. Notably, hBD-2, as well as the constitutively expressed hBD-1, exert only weak activity against *S. aureus*, whereas hBD-3 and RNase 7 are highly active against this bacterium [17]. *S. aureus* has also evolved strategies to overcome AMP action like the surface protein iron surface determinant A (IsdA), which decreases bacterial cellular hydrophobicity rendering the bacteria resistant against hBD-2 [54]. The effect of the individual AMP on growth promotion and migratory behavior of the keratinocyte seems to be largely dependent on the origin of the cell [55]. As an example, if mucosal SCC cells originating from the oral cavity are challenged with hBD-2, decreased migration and proliferation are noted, whereas mucosal SCC cells originating from the esophagus, lung, or the uterine cervix, show increased migration and proliferation due to hBD-2 challenge [13,14,56,57]. Thus, altered hBD expression and tumor promoting effects are argued to be cancer-origin specific [55]. We investigated SCC cells originating from cutaneous tumors in our experiments, which is to the best of our knowledge the first such reported experimentation. Here, only hBD-2 expression was induced via *S. aureus* challenge, whereas hBD-1 and hBD-3 were not induced. Importantly, only hBD-2 challenge promoted keratinocyte tumor cell proliferation, and none of these effects were seen in HaCaT cells, a model for non-tumorous keratinocytes. Notably, skin harbors a different microbiota composition than the oral or GI mucosa; thus, cutaneous and mucosal SCC development and the involvement of the resident microbiota might greatly differ [58]. Interestingly, induction of skin keratinocyte proliferation by *S. aureus* was recently shown also in atopic dermatitis [45]. In this non-neoplastic disease, lowered AMP expressions (e.g., hBD-2, hBD-3 and LL-37), which are driven by skewed cytokine signaling, seem to predispose for *S. aureus* colonization [59,60,61]. However, severity of infection and inflammation in atopic dermatitis again influences AMP expression levels [62]. Thus, the molecular factors of *S. aureus* to drive skin pathogenesis as well as tumorigenesis might be context-dependent and need to be elucidated in future research.

## 4. Materials and Methods 

### 4.1. Ethics Statement

The use of tissue specimens was approved by the institutional review board of the Medical University of Graz (24-167ex11/12; 25-293ex12/13).

### 4.2. Specimens, Histology, and Scoring

Formalin-fixed paraffin-embedded (FFPE) tissue specimens from total excisions (*n* = 54), punch biopsies (*n* = 24), and shave biopsies (*n* = 10) were obtained from the files of the Institutes of Dermatology and Pathology at the Medical University of Graz. The following entities were used: Actinic keratosis (*n* = 25), squamous cell carcinoma (*n* = 22), and basal cell carcinoma (*n* = 13). Additionally, non-neoplastic skin samples representing resection margins without lesions (NS; *n* = 10) and healthy skin originating from plastic surgery (HS; *n* = 13), as well as samples from chronic plaque psoriasis (PS; *n* = 5) were used for comparisons. Metadata and analyses performed on specimens are provided in Table S1. Histology scoring was performed on hematoxylin & eosin-, Gram-, and PAS-stained slides with the following parameters: microbial structures (bacteria, fungi; they were differentiated based on staining behavior in Gram and PAS stains as well as their cell size and shape): 0 = not visible, 1 = sparse, 2 = small clusters of microbial structures, 3 = abundant microbial structures; inflammation (i) % of papillary dermis infiltrated with inflammatory cells and (ii) semiquantitative (0 = none; 1 = focal; 2 = clusters of inflammatory cells in papillary dermis; 3 = dense bands of inflammatory cells in papillary dermis); neutrophilic granulocytes (no/high power field); hyper-/parakeratosis (average (mean ± SD) thickness in µm over the whole lesion).

### 4.3. DNA Isolation and Quantification

DNA extraction was optimized for isolation of DNA from FFPE skin samples. We initially compared the performance of two commercially available kits, the QIAamp DNA FFPE tissue kit (Qiagen, Hilden, Germany) and the Maxwell 16 FFPE Plus LEV DNA purification kit (Promega, Mannheim, Germany) and added sequentially beat-beating steps and additional enzymes (mutanolysin and lysostaphin) to the extraction procedure. DNA extracts were then tested with equal amounts of input DNA and by analysis with pan-bacterial and *P. acnes*-specific primers (Table S2) in a real-time PCR assay (Figure S14). To that end, the Maxwell 16 FFPE Plus LEV DNA purification kit proved superior, and the extraction procedure was as follows: (i) About 30 to 40 5 µm thick sections were cut using a new sterile blade for each FFPE tissue block, and the initial 5 sections were discarded. (ii) After deparaffinization, the pre-elute sample was transferred to Magna Lyser tubes with 1.4 mm ceramic beads (Roche Diagnostics, Mannheim, Germany) for homogenization at 6000 rpm for 30 s in a Magna Lyser centrifuge (Roche Diagnostics). (iii) Subsequently, 2.5 µL lysozyme (100 mg/mL; Carl Roth, Karlsruhe, Germany), 1.5 µL lysostaphin (4000 U/mL; Sigma Aldrich, St. Luis, MO, USA) and 3 µL mutanolysin (25,000 U/mL; Sigma Aldrich) were added to 250 µL of the homogenized sample, followed by incubation for 1 h at 37 °C [63]. (iv) Thereafter, 25 µL of proteinase K (20 mg/mL, Promega) were added to the mixture and incubated overnight at 70 °C with mild shaking (350 rpm). DNA was then purified using the automated Maxwell 16 instrument according to the manufacturer’s recommendations (Promega). DNA quality and concentration were determined spectrophotometrically with a NanoDrop ND-3300 instrument and the PicoGreen assay (Thermo Fisher Scientific, Waltham, MA, USA). 

### 4.4. 16S rRNA Gene PCR, Library Preparation and Sequencing

The V1-2 region of the 16S rRNA gene was amplified via PCR using oligonucleotide primers 27F and 357R (Table S2). All PCR reactions were performed in triplicates with 5 µL of input DNA (~15 ng/µL) containing 1× Fast Start High Fidelity Buffer (Roche), 1.25 U High Fidelity Enzyme (Roche), 200 µM dNTPs (Roche), 10 pmol primers, and PCR-grade water (Roche) to a final volume of 25 µL. PCR cycling was performed as described previously [64]. Triplicates from each sample were pooled, and the amplicons were checked visually on 1% agarose gels; 15 µL of the pooled PCR products were then normalized on a SequalPrep Normalization Plate (Life Technologies, Vienna, Austria) according to the manufacturer’s instructions. Then 15 µL of the normalized products were used as template for indexing PCR in a total volume of 50 µL to introduce barcode sequences as described [65]. Cycling conditions were the same as for the 16S rRNA gene target with only eight cycles of amplification [64]. After indexing, 5 µL of each sample were pooled, and a total of 50 µL were loaded onto 1% agarose gels and subsequently purified from the gel with the Qiaquick Gel Extraction Kit (Qiagen, Hilden, Germany). DNA was quantified using PicoGreen dsDNA reagent (Life Technologies) according to the manufacturer’s instructions and size-validated on an Agilent 2100 Bioanalyzer (Agilent Technologies, Santa Clara, CA, USA). The sequencing library was resolved on a MiSeq desktop sequencer (Illumina, Eindhoven, Netherlands) according to the manufacturer’s recommendations.

### 4.5. Microbiota and Correlation Analysis

Three negative controls (paraffin blank extraction, kit blank extraction, and 16S rRNA gene PCR no-template control) were amplified and sequenced along with our sample set and subjected to the same procedure of microbiota analysis. Raw files derived from Illumina MiSeq were processed according to the standard MiSeq SOP of MOTHUR v.1.33.3. [66]. Sequencing errors were reduced using MOTHUR’s pre.cluster command to remove sequences that arose due to sequencing errors [67]. Chimeras were removed with UCHIME v.1.22 [68], and non-bacterial contaminants such as chloroplasts and mitochondria were removed by classify.seqs and remove.lineage using the Ribosomal Database Project (RDP) training set v.9 [69]. The high-quality reads were aligned to the SILVA database v.119 [70,71]. For operational taxonomic unit (OTU)-based analyses, the processed fasta files from MOTHUR were introduced into QIIME v.1.8.0 [72]. OTUs were formed by clustering the sequences with UCLUST [73], with 97% similarity (OTU97%ID), and taxonomy was assigned by using the RDP classifier and Greengenes reference v.13.8. A de novo OTU picking strategy was employed. Subsequently, diversity analyses were performed in QIIME according to the core_diversity_analysis.py workflow. For statistical comparisons of alpha-diversity metrics, unpaired *t*-tests with 999 Monte Carlo permutations were performed, and the Bonferroni method was used for multiple comparison corrections. Calculated beta diversity metrics (weighted unifrac; [74]) were compared by using the nonparametric ANOSIM measure. Significant differences in relative abundances of taxa were calculated by using a nonparametric Kruskal–Wallis test using false discovery rate (FDR) correction and linear discriminant analysis effect size (LEfSe) v.1.0 [75]. *p*-values ≤ 0.05 were considered statistically significant (* *p* < 0.05; ** *p* < 0.01; *** *p* < 0.001). Presented values are always mean ± SD if not indicated otherwise. A batch file specifying the analysis parameters is given in the supplemental material (Batch file, supplemental material). Heatmaps were constructed based on the log(10)-transformed abundance data obtained from OTUs covering more than 1% overall abundance. Species level taxonomy was assigned by using the EzBioCloud database [15]. Visualization and hierarchical clustering (hclust) were implemented in R using the heatmap.2 function provided by the gplots_3.0.1.1 package. Pearson correlation was used to calculate associations between assessed features which were computed in R 3.4.4 using method corr.test implemented in package psych vers.1.8.12 [76]. The *p*-values were corrected using Holm correction to adjust for multiple comparison test [77].

### 4.6. Quantification of Bacterial Load, Staphylococcus, S. aureus, and S. epidermidis Abundance via qPCR

Real-time PCR was employed to determine bacterial load and *Staphylococcus*, *S. aureus,* and *S. epidermidis* absolute abundance. Oligonucleotide primer sequences are listed in Table S2; 20 ng of total DNA were used as normalized input for each PCR amplification using SYBR Green PCR core reagents according to the supplier’s specifications (Applied Biosystems, Waltham, USA). PCR was performed with an ABI PRISM 7900HT instrument (Applied Biosystems). PCR cycling consisted of 10 min at 95 °C, followed by 40 cycles of 15 s at 95 °C, annealing for 1 min at 55 °C for panbacterial and *Staphylococcus*-specific PCR; at 62 °C for the *S. aureus*-specific PCR; or at 60 °C for the *S. epidermidis* PCR, respectively, and finally, 15 s at 95 °C, 1 min at 60 °C, and 15 s at 95 °C. Each qPCR analysis was performed in triplicate and repeated three times.

### 4.7. Reverse Transcription Quantitative PCR (RT-qPCR) 

Total RNA from FFPE samples (10 sections, each 5 µm thick) was isolated with deparaffinization solution (Qiagen) and the RNeasy FFPE kit, which includes a DNase treatment step (Qiagen). For cDNA synthesis, 200 ng of total RNA was used with the GeneAmp RNA PCR kit according to the manufacturer’s recommendations (Thermo Fischer Scientific). RNA quality and quantity were determined spectrophotometrically using a NanoDrop instrument (ThermoScientific). qPCR was performed with an ABI PRISM 7900HT instrument (Applied Biosystems) and the SYBR Green PCR core reagents (Applied Biosystems). Reaction mixtures were set up as described above. Each PCR reaction was performed in triplicate. RNA from cell culture experiments were extracted using PeqGOLD total RNA Kit (Peqlab, Erlangen, Germany) and transcribed into cDNA with the Transcriptor First Strand cDNA Synthesis Kit (Roche Life Science, Indianapolis, IN, USA) according to the manufacturer’s instructions. qRT-PCR for these samples was performed using SYBR green PCR master mix (Sigma) on the StepOnePlus Real-Time PCR system (Applied Biosystems). The oligonucleotide primer sequences for antimicrobial peptides (hBD-1, -2, -3, RNase 7, and Psoriasin) are shown in Table S2. For each mRNA target, the expression level was normalized using human beta-actin as a reference and quantified via the 2-ΔΔCt method [78].

### 4.8. Fluorescent In Situ Hybridization (FISH)

FISH was performed with modifications as described previously [79,80]. Briefly, 5 µm sections of skin tumor samples were mounted on adhesive superfrost slides (DAKO FLEX slides; Sigma Aldrich). The slides were first incubated at 70 °C for 1 h in a non-CO2 incubator followed by 10 min incubation twice in xylene (Sigma Aldrich) for deparaffinization. Subsequently, samples were dehydrated by incubation in ethanol with increasing concentrations (50%, 80%, and 99%, respectively; 3 min each step). Before hybridization, each section was pretreated with 20 mg/mL lysozyme (Carl Roth) and incubated for 30 min at 37 °C, followed by incubation in 1 mg/mL lysostaphin (Sigma Aldrich) for 30 min at 37 °C [81]. The reaction was stopped by incubation in absolute methanol for 1 min. Control slides contained fixed E. coli, *S. epidermidis*, and *S. aureus* cells derived from bacterial culture. For probe hybridization, samples were incubated in hybridization buffer containing 20% (*v/v*) formamide (PanReac AppliChem, Barcelona, Spain), 20 mmol/L Tris-HCl, pH 7 (AMRESCO, OH, USA), 2% (*w/v*) sodium dodecyl sulfate (Sigma), 0.9 M NaCl (VWR Chemicals, Pennsylvania, USA) for 15 min at 46 °C, followed by addition of a mixture of probes (50 ng each); Cy3-labeled EUB338/I probe [82] directed against bacteria and a Cy5-labeled Sau probe for *S. aureus* [83]. A NONEUB-probe (Eurofins) was used as a nonsense negative control and specificity of the *S. aureus* probe was verified by hybridization of mixed cultures made of *S. epidermidis* and *S. aureus* (Figure S15). Probe sequences are given in Table S2. The slides were transferred to a humid chamber and incubated at 46 °C for 2 h. Subsequently, slides were washed in a buffer containing 10 mM Tris/HCl (pH 7), 0.225 M NaCl, and 2% sodium dodecyl sulfate at 46 °C for 15 min. Thereafter, slides were washed with cold water and allowed to dry at room temperature before being counterstained with DAPI (Sigma Aldrich). Slides were analyzed using a Zeiss LSM 510 confocal microscope (Carl Zeiss, Jena, Germany) using the following filter settings (excitation/emission): DAPI—358 nm/463 nm, CY3—549 nm/562 nm, and CY5—646 nm/664 nm. FISH signals were scored according the following system: 0 = no signal, 1 = single signals, 2 = small groups of signals, 3 = large clusters of signals.

### 4.9. Cell Culture, Infection Assay, and Flow Cytometry

*S. aureus* strains (DSM799, DSM11823) and *S. epidermidis* (Hyg 9209-15) were routinely cultured under aerobic conditions on Columbia blood agar plates (BioMerieux, Marcy l’Etoile France) at 37 °C for 24 h. Cells from the human cutaneous squamous cell carcinoma cell lines HSC-1 and SCL-1 [84,85] and the human keratinocyte cell line HaCaT [86] were seeded in six-well plates at a density of 2 × 10^5^ cells per well in 2 mL of Dulbecco’s modified Eagle’s medium (DMEM) low glucose (1 g/L) (GE Healthcare, Vienna, Austria) and 10% fetal bovine serum (FBS) (Thermo Fischer Scientific). The cells were grown to 80% confluence in a water-saturated atmosphere of 95% air and 5% CO_2_ at 37 °C. Prior to the infection, a single *S. aureus* colony was inoculated into a 100 mL culture flask containing 30 mL DMEM low glucose (1 g/L) and 1.5% FBS and incubated with gentle agitation (160 rpm) at 37 °C for 14 h. Subsequently, cells were infected at a multiplicity of infection (MOI) of 1:2, 1:20, and 1:50, respectively for 24 h [53]. After 24 h, cells were collected for RNA isolation in addition to the supernatant to assess the *S. aureus* load after 24 h of infection by bacterial colony forming units (CFU) plating via serial dilution on Columbia blood agar plates (BioMerieux). In parallel cell numbers, viability and apoptosis of cells were determined by flow cytometry. Briefly, HSC-1, SCL-1, and HaCaT cells were harvested after 24 h infection using trypsin for assessment of cell viability and apoptosis. 7-AAD viability staining solution (eBioscience, CA, USA) or the Annexin V Apoptosis Detection Kit APC (eBioscience) was used following the manufacturer’s protocol. Cells were analyzed using a CytoFLEX flow cytometer (Beckman Coulter, California, USA), and results were calculated using CytExpert Software (Beckman Coulter) [87]. Each experiment was repeated three times. 

### 4.10. CCK-8 Assay

HSC-1 and SCL-1 cells were plated in a 96-well plate at a density of 1 × 10^4^ cells/well in a water-saturated atmosphere of 95% air and 5% CO_2_ at 37 °C overnight. The cells were then treated with 20 µg/mL of hBD-1, -2, and -3 for up to 24 h. Subsequently, proliferation was assessed using the CCK-8 kit (Sigma-Aldrich) according to a previously described protocol [13]. CCK-8 solution was added to each well after treatment and incubated for 2 h. The absorbance was measured at 450 nm using a SPECTROstar Omega microplate reader (BMG Labtech, Offenburg, Germany). 

### 4.11. XCELLigence^TM^ Real-Time Cell Proliferation Assay 

The xCELLigence^TM^ Real-Time Cellular Analysis (RTCA) system (Roche and ACEA Biosciences, California, USA) was used to monitor cell proliferation using impedance as the read out. The impedance measurement, displayed as cell index (CI) takes cell number, viability, and morphology into account; 1 × 104 HSC-1 or SCL-1 cells were treated with 20 µg/mL hBD-1, -2, and -3 (PeptaNova, Sandhausen, Germany) [13], respectively, and seeded in DMEM low glucose (1 g/L) with 1.5% FBS in E-plates 16 (ACEA Biosciences). Proliferation was monitored every 15 min by the xCELLigence system [76] for up to 24 h. Growth curves were normalized to the time point of cell adherence (~4 h). Evaluations were performed using xCELLigence 1.2.1 software (ACEA Biosciences). Each experiment was repeated three times.

### 4.12. Statistical Analysis 

Quantitative PCR and cell proliferation data were assessed with the Shapiro–Wilk test for normality distribution. Data are given as mean ± SEM if not otherwise specified, and statistical testing was performed with GraphPadPrism 5. *p*-values < 0.05 were considered statistically significant.

### 4.13. Availability of Data and Material

The sequencing data generated for this study are available in the EBI short read archive with the accession number PRJEB23563.

## 5. Conclusions

In summary, our findings point out that changes in skin microbiota emerging during skin carcinogenesis could promote tumor cell growth via modulation of AMP expression. A putative model summarizing our findings is highlighted in Figure S16. To that end, analyzing the contribution of the skin microbiota to tumorigenesis will allow for a better understanding of the complex interplay between the tumor microenvironment and tumor cells. This knowledge might open new avenues for risk assessment and innovative therapies modulating the microbiota in UV-damaged skin, thus counteracting SCC development and progression [8].

## Supplementary Materials

The following are available online at https://www.mdpi.com/2072-6694/12/3/541/s1, Table S1: Specimen designations and analyses performed, Table S2: Oligonucleotide primers and probes [88,89,90,91,92,93,94,95,96], Table S3: Differential abundant taxa revealed by LEfSe (keratinocyte tumors), Table S4: Differential abundant taxa revealed by LEfSe (keratinocyte tumors and healthy skin), Figure S1: Hyper- and parakeratosis is associated with AK and SCC, Figure S2: Microbial structures are associated with the lesional surface and with invasive tumor tissue, Figure S3: Different microbial community types in keratinocyte skin tumors and healthy skin, Figure S4: Differential abundant taxa in keratinocyte tumors (log(10)-transformed read data), Figure S5: LEfSe bar-chart output (keratinocyte tumors), Figure S6: LEfSe output as in Figure 2H with higher magnification, Figure S7: LEfSe bar-chart output (keratinocyte tumors and healthy skin), Figure S8: LEfSe output as in Figure S3GH with higher magnification, Figure S9: Heat map representation of dominant taxa revealed by unsupervised hierarchical clustering, Figure S10: Significant increase of *S. aureus* loads in SCC compared to its adjacent non-lesional skin, Figure S11: qPCR (bacterial load, *Staphylococcus* and *S. aureus* loads) including psoriasis samples, Figure S12: CFU plating of *S. aureus* strains after co-culture, Figure S13: Viable cells, apoptosis, and necrosis assay by flow-cytometry, Figure S14: Optimization of DNA extraction out of FFPE specimens, Figure S15: Specificity testing of the *S. aureus*-specific FISH probe, Figure S16: Scheme indicating the proposed model of SCC tumorigenesis, Batch file: Batch file specifying microbiota analysis parameters.
