# Supplementary material for: Molecular Profiling of Keratinocyte Skin Tumors Links Staphylococcus aureus Overabundance and Increased Human β-Defensin-2 Expression to Growth Promotion of Squamous Cell Carcinoma
Source: Cancers (Basel). 2020 Feb 26;12(3):541. doi: 10.3390/cancers12030541 (PMC7139500; doi:10.3390/cancers12030541)
Supplement: Supplementary file 1 [file cancers-12-00541-s001.zip › cancers-650819-supplementary-final check 2/cancers-650819-supplementary-final check 2.docx]

Supplementary Materials

Molecular Profiling of Keratinocyte Skin Tumors Links *Staphylococcus aureus* Overabundance and Increased Human β-Defensin-2 Expression to Growth Promotion of Squamous Cell Carcinoma

Nandhitha Madhusudhan, Manuela R. Pausan, Bettina Halwachs, Marija Durdević, Markus Windisch, Jan Kehrmann, VijayKumar Patra, Peter Wolf, Petra Boukamp,
Christine Moissl-Eichinger, Lorenzo Cerroni, Jürgen C. Becker and Gregor Gorkiewicz


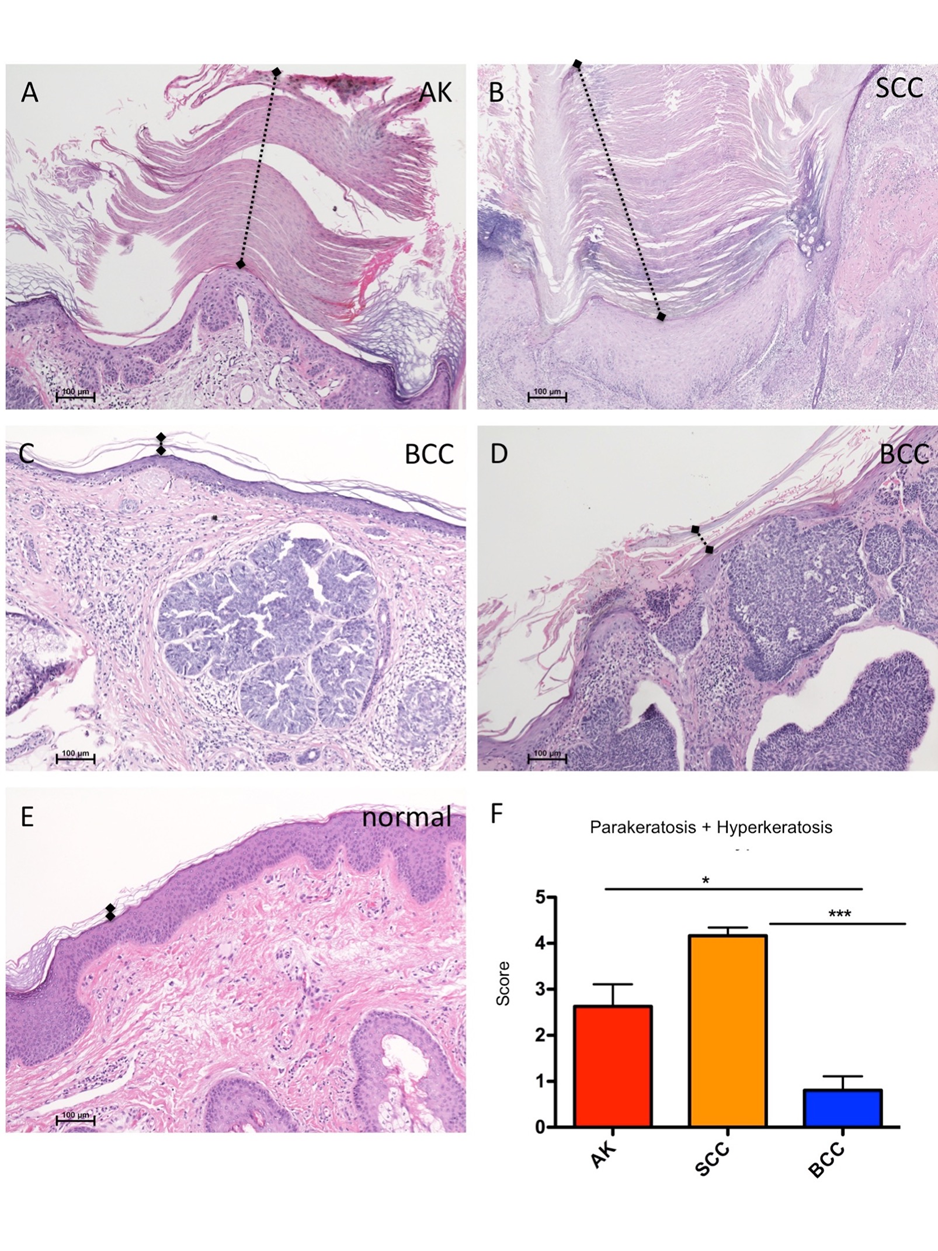


**Figure S1.** Hyper- and parakeratosis is associated with AK and SCC. The keratin layer is marked with a dashed line. Note the increased keratin layer in AK (**A**) and SCC (**B**). BCC shows no or just sparse hyper-/parakeratosis (**C,D**)**.** A normal skin (originating from the chest) is shown for comparison **(E)**. **(F)** Microscopic scoring of hyper- and parakeratosis indicates predominance in AK and SCC (* *p* < 0.05; *** *p* < 0.005, Kruskal Wallis test; Dunn’s multiple comparison test; AK, *n* = 12; SCC, *n* = 12; BCC, *n* = 13).


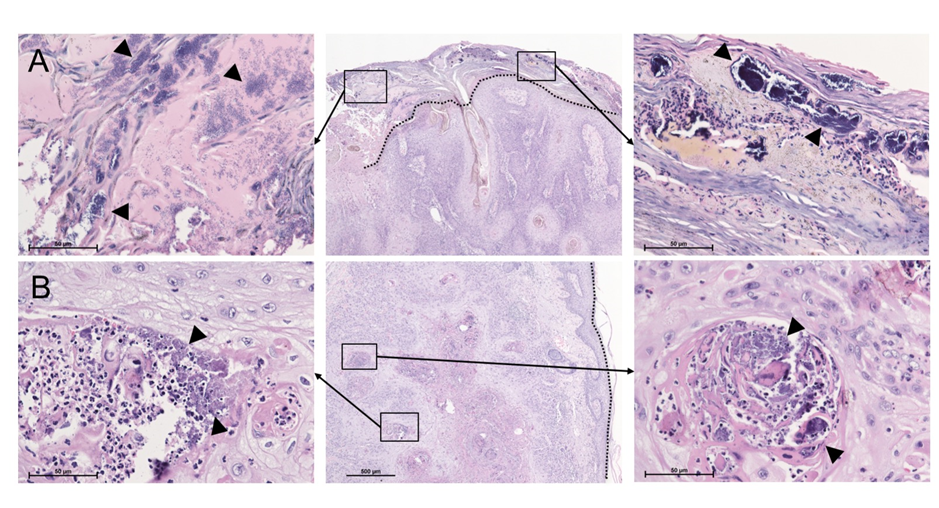


**Figure S2.** Microbial structures (arrow heads) are associated with the lesional (**A**) but also with invasive tumor tissue (**B**) in SCC**.** The dashed lines specify the skin surface.


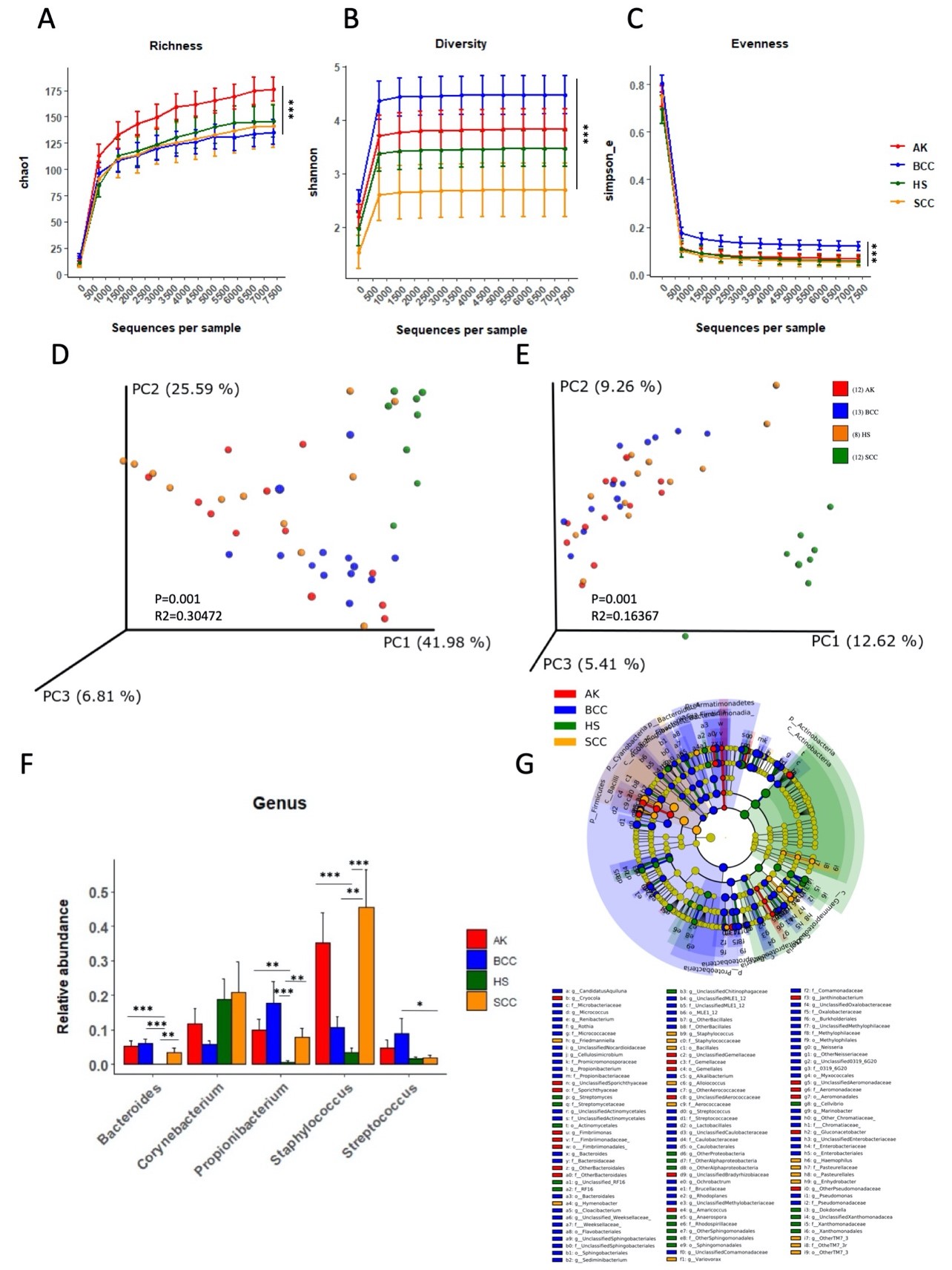


**Figure S3.** Different microbial community types in keratinocyte skin tumors and healthy skin. **(A)** Microbial richness (Chao1) **(B)** diversity (Shannon) and **(C)** evenness (Simpson) were significantly different between all groups (Kruskal-Wallis rank sum test; *p* < 0.0001). **(D**–**E)** Principal coordinate analysis indicates significantly different microbial communities in all entities (measure: D, weighted unifrac; E, unweighted unifrac; ANOSIM). **(F)** Significantly different relative abundance of *Staphylococcus*, *Streptoccocus, Propionibacterium* and *Bacteroides* (Dunn's post hoc test, Benjamini-Hochberg correction; ** *p* < 0.01; *** *p* < 0.005). **(G)** LEfSe analysis specifying taxa with increased relative abundance in tumors (a comprehensive bar-chart and tabular representation of the LEfSe output is given in Figure S7 and Table S4; a higher magnification of Figure S2G is given as Figure S8).


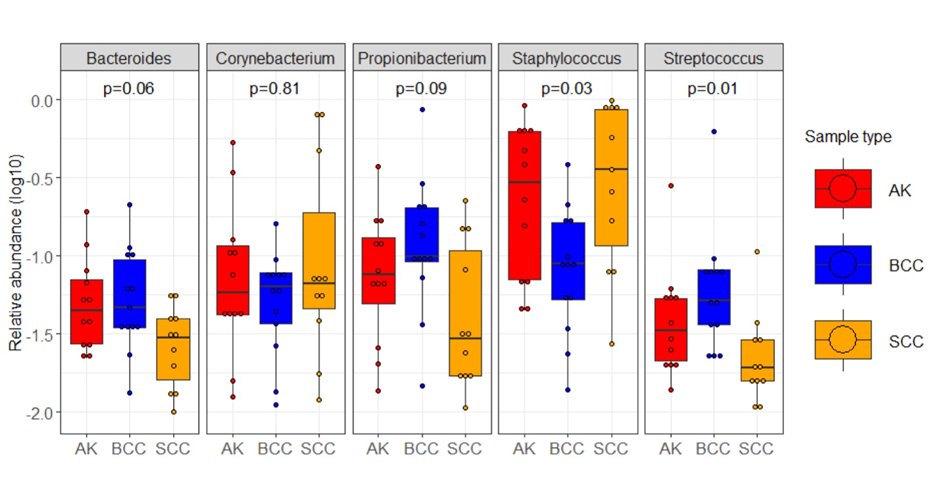


**Figure S4.** Differential abundant taxa in keratinocyte tumors (log(10)-transformed read data).


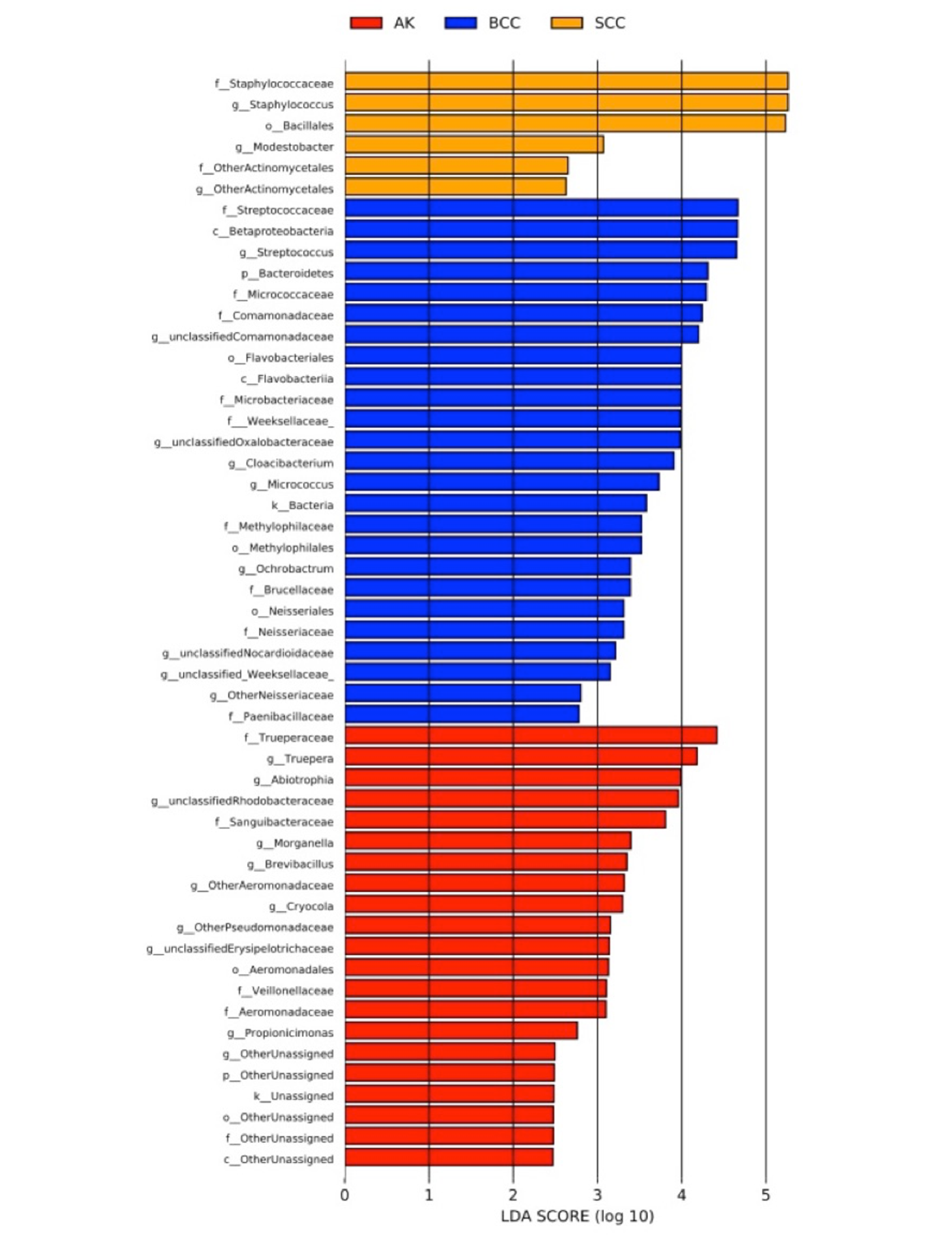


**Figure S5.** LEfSe bar-chart output (keratinocyte tumors).


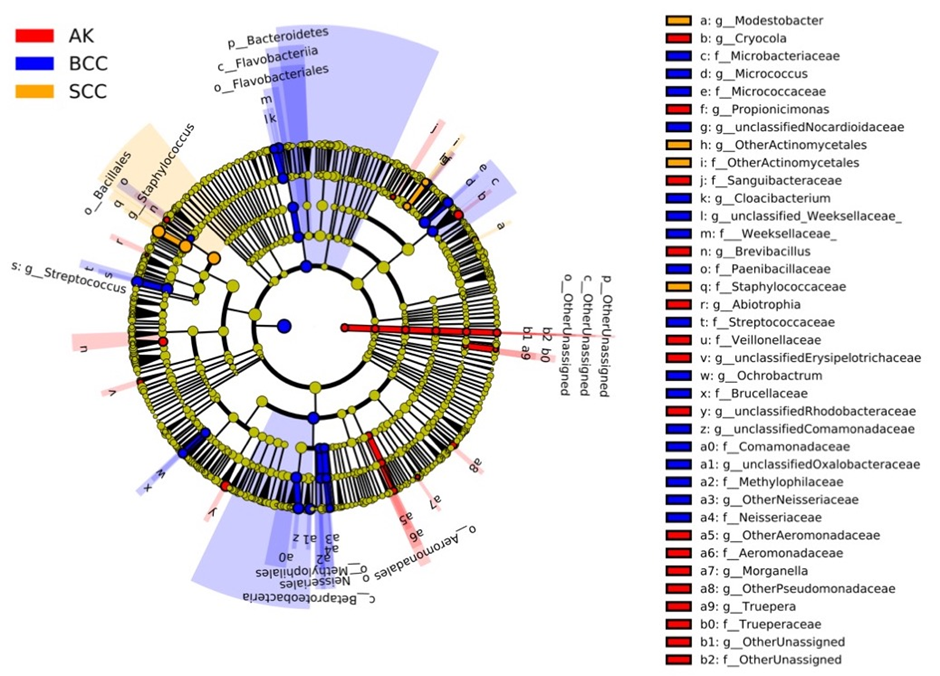


**Figure S6.** LEfSe output as in Figure 2H with higher magnification.


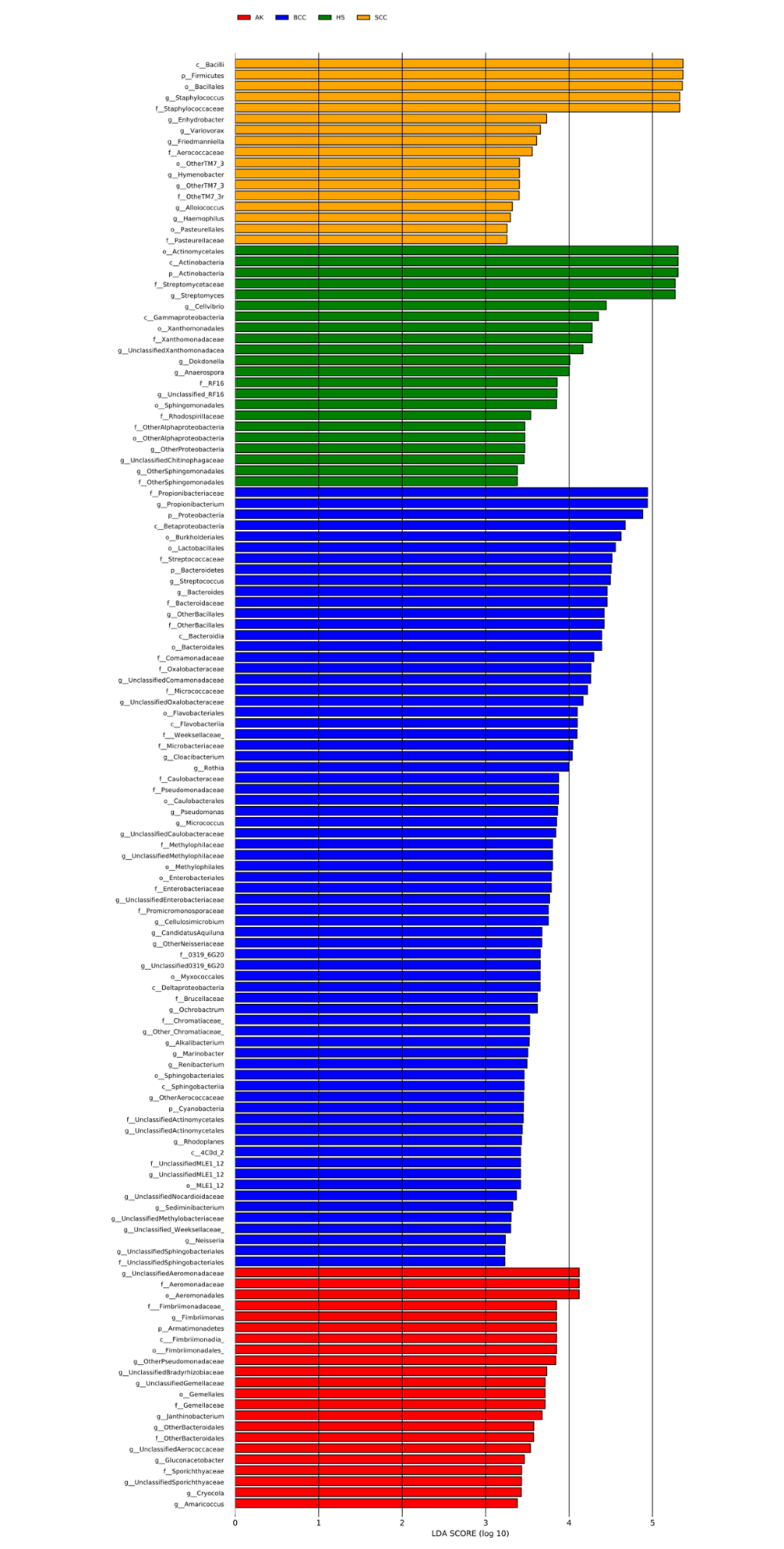


**Figure S7.** LEfSe bar-chart output (keratinocyte tumors and healthy skin).


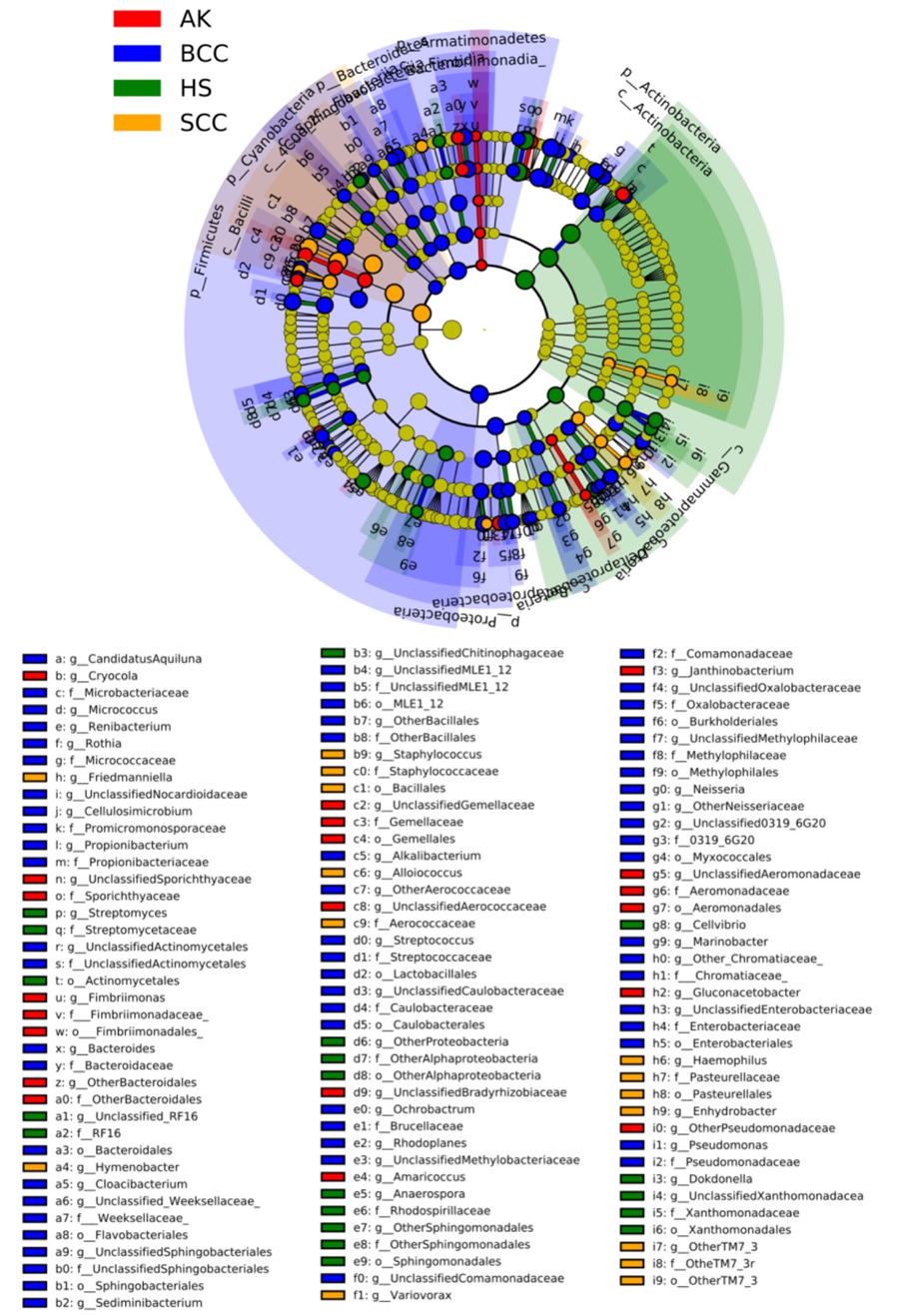


**Figure S8.** LEfSe output as in Figure S3GH with higher magnification.


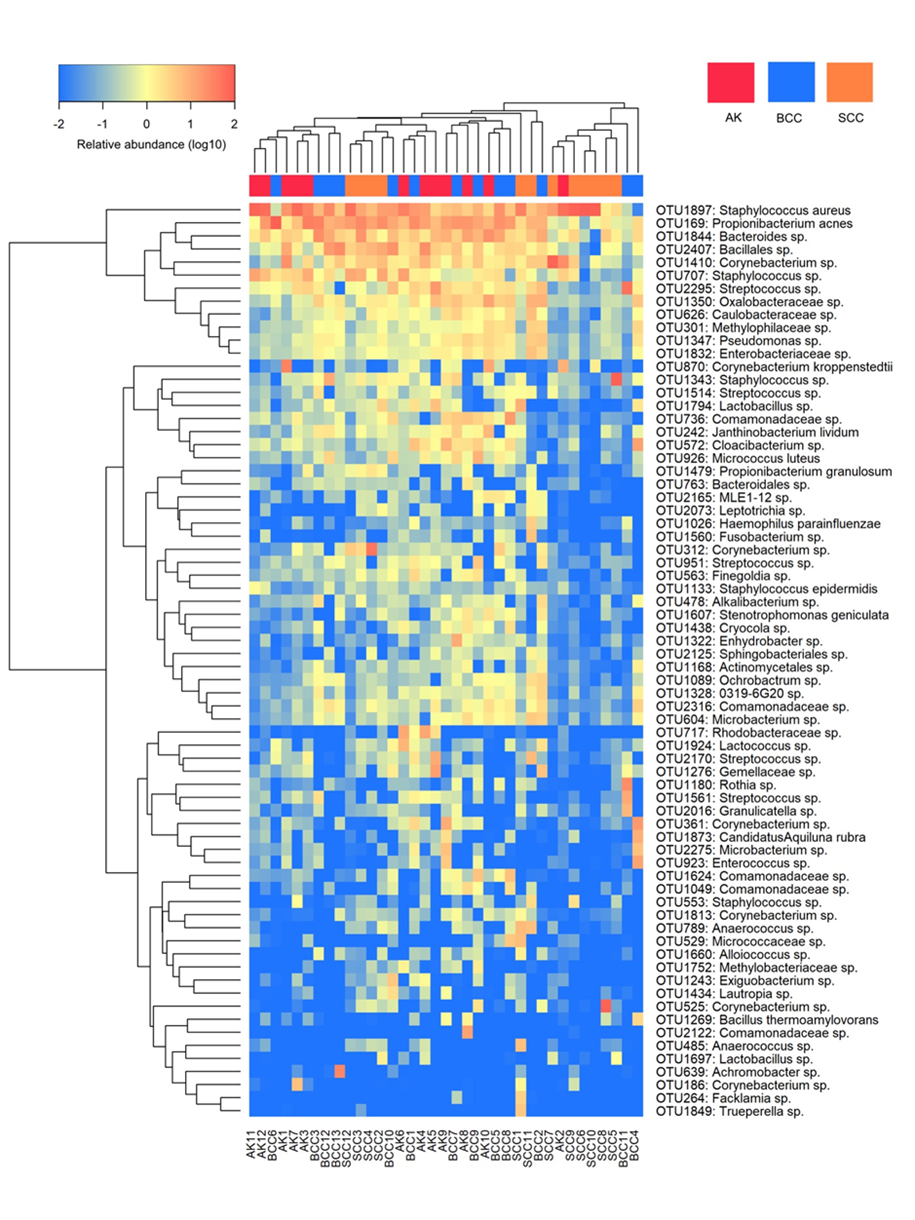


**Figure S9.** Heat map representation of dominant taxa revealed by unsupervised hierarchical clustering.


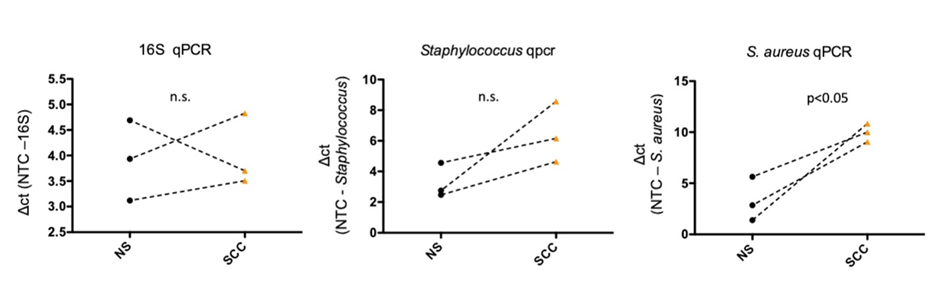


**Figure S10.** Significant increase of *S. aureus* abundance in cSCC compared to its adjacent non-lesional skin (NS) as assessed by qPCR in paired samples (*p<0.05; paired t-test; n=3).


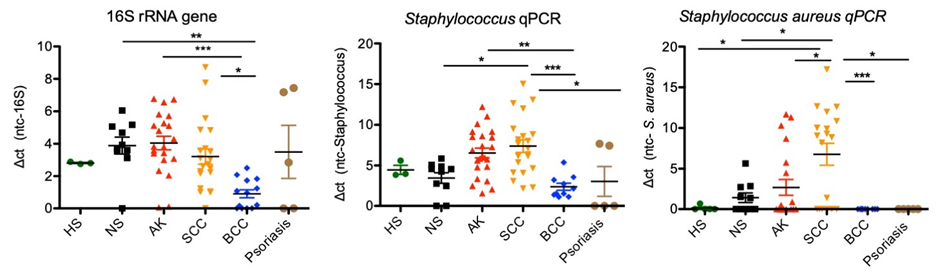


**Figure S11.** qPCR (bacterial load, *Staphylococcus* and *S. aureus* loads) including psoriasis samples qPCR (bacterial load, *Staphylococcus* and *S. aureus* loads) including psoriasis samples (ANOVA; Turkey’s multiple comparison test; * *p* < 0.05; ** *p* < 0.01; *** *p* < 0.005).


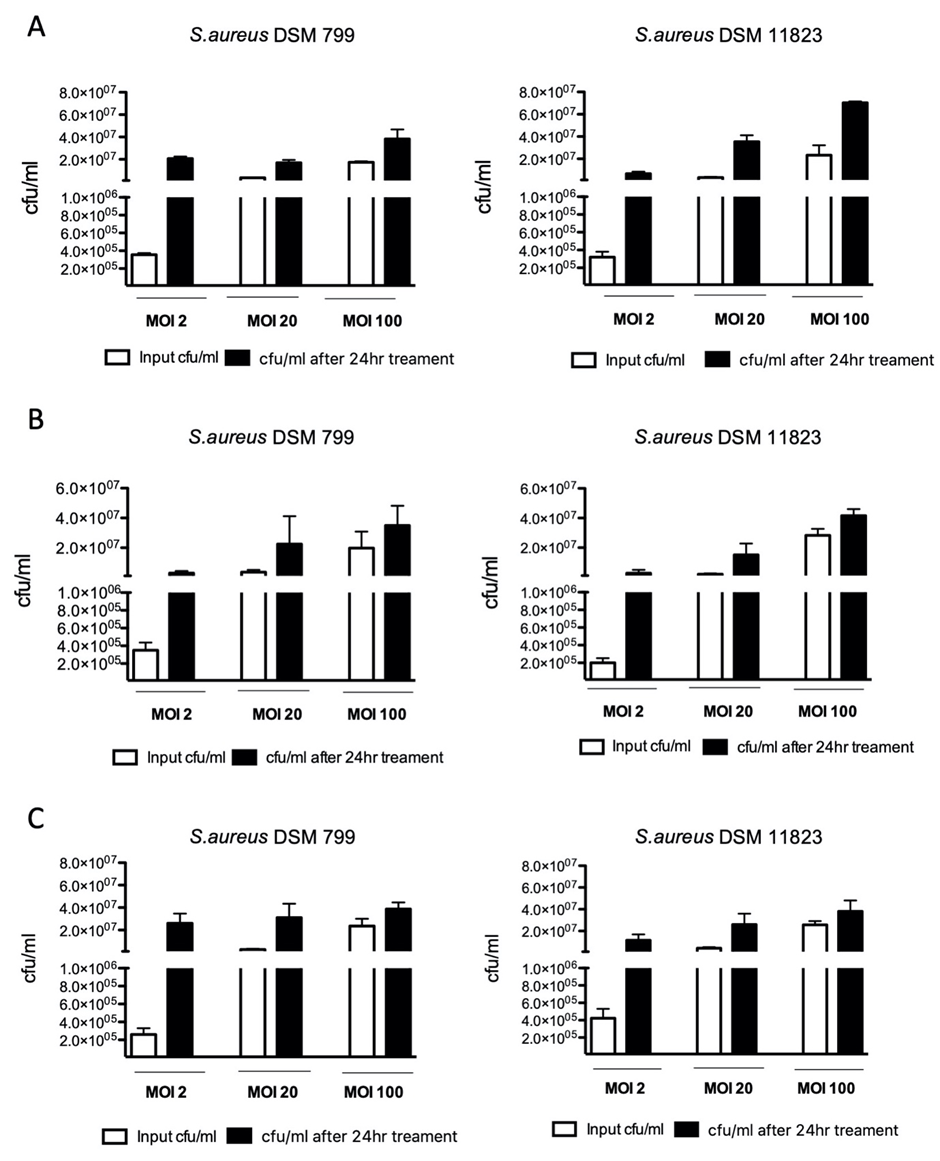


**Figure S12.** CFU plating of *S. aureus* strains after co-culture.


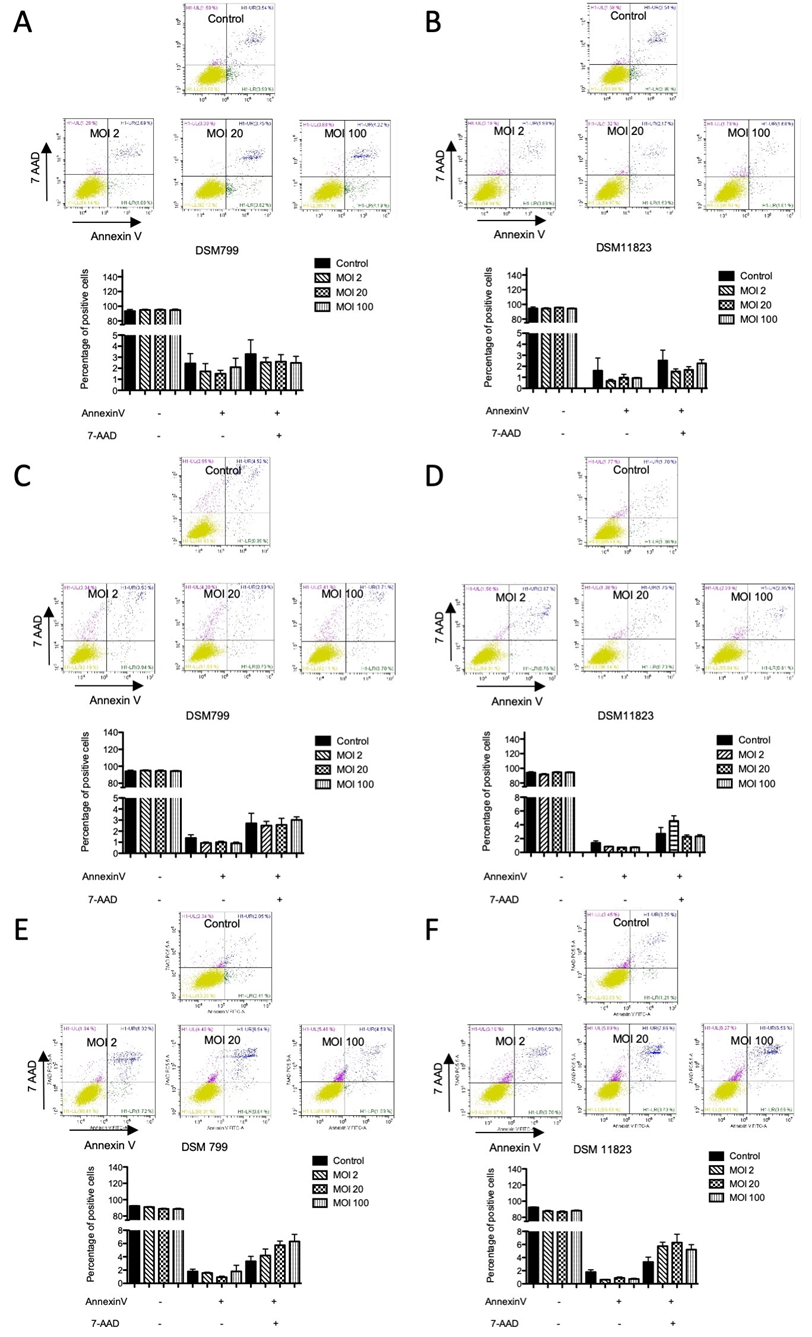


**Figure S13.** Viable cells, apoptosis, and necrosis assay by flow-cytometry (**A**,**B**), SCL-1 (**C**,**D**) and HaCaT (**E**,**F**) cells challenged with *S. aureus* DSM799 and DSM11823. No significant difference in apoptotic and necrotic cells were evident compared to controls.


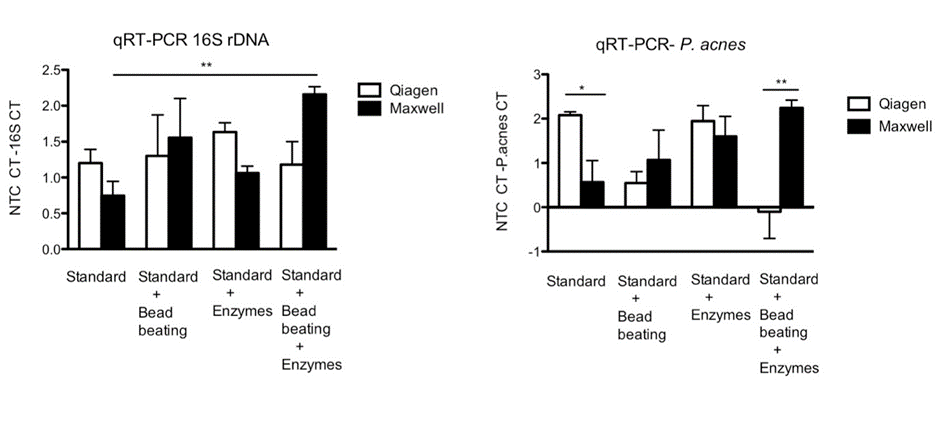


**Figure S14.** Optimization of DNA extraction out of FFPE specimens comparing two commercially available kits and adding sequentially a beat-beating step and enzymes. Real-time PCR was used to determine DNA yield and amplification by using pan-bacterial and *P. acnes* specific primers (see Table S2 for sequences; *n* = 3, * *p* < 0.05, ** *p* < 0.01, one-way ANOVA, Bonferroni correction).


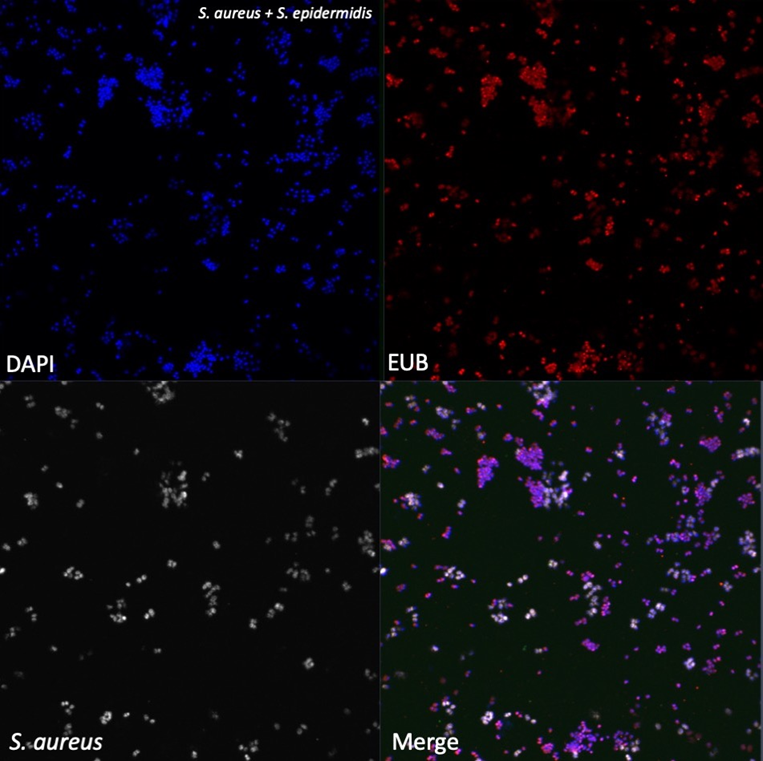


**Figure S15.** Specificity testing of the *S. aureus*-specific FISH probe. Mixed cultures of *S. aureus* and *S. epidermidis* were stained with (**A**) DAPI, (**B**) the panbacterial prob (EUB) and (**C**) a *S. aureus* specific probes. The *S. aureus* probe specifically stains *S. aureus* cells (**C**) and (**D**).


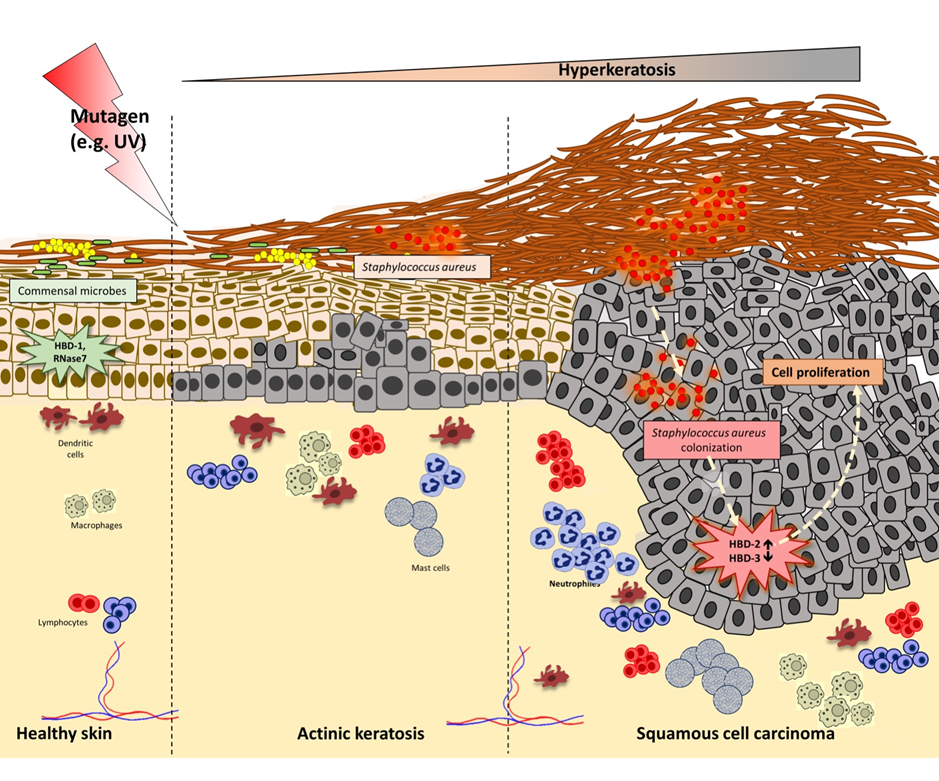


**Figure S16.** Scheme indicating the proposed model of SCC tumorigenesis, Batch file: Batch file specifying microbiota analysis parameters. Neoplastic transformation (e.g., via UV) leads to an altered microbial habitat (e.g., via increased keratin production leading to hyperkeratosis). This favors protumorigenic microbiota, specifically high loads of *S. aureus*, which modulate expression of hBDs. Induced hBD-2 confers a protumorigenic growth stimulus on tumor cells, thereby promoting tumor growth.

| 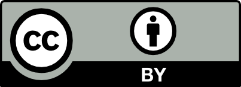 | © 2020 by the authors. Licensee MDPI, Basel, Switzerland. This article is an open access article distributed under the terms and conditions of the Creative Commons Attribution (CC BY) license (http://creativecommons.org/licenses/by/4.0/). |
| --- | --- |
